# A noncoding RNA containing a SINE-B1 motif associates with meiotic metaphase chromatin and has an indispensable function during spermatogenesis

**DOI:** 10.1371/journal.pone.0179585

**Published:** 2017-06-28

**Authors:** Ryusuke Nakajima, Takuya Sato, Takehiko Ogawa, Hideyuki Okano, Toshiaki Noce

**Affiliations:** 1 Department of Physiology, Keio University School of Medicine, 35 Shinamomachi, Shinjuku-ku, Tokyo, Japan; 2 Laboratory of Proteomics, Institute of Molecular Medicine and Life Science, Yokohama City University Association of Medical Science, Yokohama, Kanagawa, Japan; China University of Science and Technology, CHINA

## Abstract

A search for early response genes that are activated following germ cell induction from mouse embryonic stem cells *in vitro* led us to the isolation of a long noncoding RNA that contains a SINE (short interspersed element)-B1F motif that was named *R53*. *In situ* hybridization and northern blot analyses revealed that the *R53* subfragment RNA bears a B1F motif, is processed from the primary transcript, is expressed in adult testis and is predominantly localized in meiotic metaphase chromatin during spermatogenesis. Recent studies of chromosome-associated RNAs have explored novel functions of noncoding RNAs. Specifically, chromosome-bound noncoding RNAs function not only as structural components of chromosome but also as scaffolds that recruit epigenetic modulators for transcriptional regulation, and they are dynamically rearranged during the cell cycle. However, few studies have explored meiotic chromatin; thus, *R53* RNA appears to be the first long noncoding RNA to be tightly associated with the metaphase chromatin during spermatogenesis. Furthermore, *R53* knockdown using a lentivirus-mediated RNAi injected into mouse testis and organ culture of the fragments revealed a remarkable reduction in postmeiotic cells and irregular up-regulation of several postmeiotic genes, which suggests the possibility that the SINE-B1-derived noncoding RNA *R53* plays an indispensable role in the transcriptional regulation of key spermatogenesis genes.

## Introduction

Recent investigations have revealed that transposable element (TE) inserts, which account for nearby 50% of most eukaryotic genomes, are not ‘junk’ or ‘parasite’ DNA but rather have great potential to cause drastic changes in the developmental pattern of gene expression by increasing genetic variation and the recombination rate [1–3]. Therefore, TEs are now regarded as a major driving force in lineage-specific and adaptive evolution [1, 4, 5]. SINEs are one of the major classes of TEs, which also include long interspersed elements (LINEs) and retrovirus-like long terminal repeat (LTR). More than 10^6^ of SINEs are scattered throughout the mammalian genome (approximately 10% of the total genomic sequence) [6–8]. SINEs originate from within small RNAs, such as 7SLRNA (cytoplasmic signal recognition particle), rRNA and tRNA, and unlike LINEs and LTRs, SINEs have their own internal RNA polymerase III (polIII) promoter [6]. Some SINE sequences have been demonstrated to function in gene expression and chromosome organization at the insertion site via the internal binding sites for various transcriptional factors (TFs) and epigenetic modifiers [2, 3, 8–12].

In addition to the functions of SINEs as *cis-*elements in the genome, SINE-derived RNA transcripts function as *trans*-elements. Pol III-transcribed SINEs are induced in cells that are exposed to stress stimuli. This response is thought to be involved in the translational regulation of SINE-containing mRNAs [6]. Additionally, many SINE-containing RNAs are transcribed by pol II as parts of pre-mRNAs, and processed SINE RNAs are abundant in the nucleus [2, 6, 13, 14]. In contrast, a recent analysis that employed deep transcript sequencing identified a number of SINE-containing noncoding RNAs, most of which are classified as long noncoding RNAs (lncRNAs) with more than 200 nt or large intergenic noncoding RNAs (lincRNA, >500 nt) [6]. In general, small noncoding RNAs of less than 100 nt (such as miRNAs, piRNAs and siRNAs), which are generated through post-transcriptional processing of their precursors, have been revealed to exert their functions through sequence-specific binding to target molecules. In contrast, lncRNAs and lincRNAs are assumed to function through their specific secondary structures to form complexes with their partner proteins, such as TFs and chromosome-associated RNA binding proteins (RBPs) [9]. Furthermore, it is of great interest that lncRNAs are a major component of interphase chromosomes and play important roles in the control of cell-type specific gene expression and chromatin stability [11, 15–17].

High-level expression of SINE-containing RNAs has been found in mouse testis, zygotes and early-stage embryos [13]. Among these RNAs, BC1 RNA, which a member of SINE-ID family provides an exceptional example of a well-demonstrated function: BC1 RNA acts as a translational regulator in both postsynaptic microdomains in the brain and in pre-meiotic spermatogonia in the testis [18, 19]. However, whether the testicular expression of SINE-containing noncoding RNAs is functional and/or relevant to spermatogenesis remains largely unknown. In this study, using *in vitro* derivation of germ cells from mouse embryonic stem cells (ESCs), we found the novel SINE-B1-containing noncoding RNA *R53*, the expression of which was induced at the onset of germ cell differentiation. In mice, the SINE-B1 family is the largest (approximately 5x10^5^ copies) of the six SINE-families (B1-4, MIR and ID). Whereas the approximately140 bp B1-motif and *Alu* elements in primates originated from the common ancestor of 7SL RNA, the nucleotide sequences of SINE-B1 copies are not identical; rather, they vary by up to 35% [6, 20]. Although transcription of SINE-containing RNAs in the testis is not unique [2, 13, 21], it was quite surprising that *in situ* hybridization (ISH) analysis revealed that the *R53* transcripts were specifically localized in the metaphase chromatin of meiotically dividing cells. Recent findings that some lncRNAs derived from LINEs and SINEs are involved in the functional constitution of higher-order structures of interphase and mitotic phase chromosome [17, 22] suggest a functional involvement of *R53* noncoding RNA in meiotic cell division in the testis. Indeed, *R53* knockdown experiments using a lentivirus-based short hairpin RNA (shRNA) vector revealed that inhibition of *R53* RNA expression results in a precocious up-regulation of several spermiogenesis-specific genes and a severe inability to produce postmeiotic germ cells. These results indicate that the SINE-B1-containing noncoding *R53* RNA has potential roles in regulating spermiogenic genes and in the control of meiotic division leading to sperm formation, which should provide novel insight to elucidate developmental roles of SINE-derived RNAs during meiosis and spermatogenesis.

## Results

### Isolation of the transcript of *R53*, a noncoding RNA containing a SINE-B1F motif

**Fig 1:**
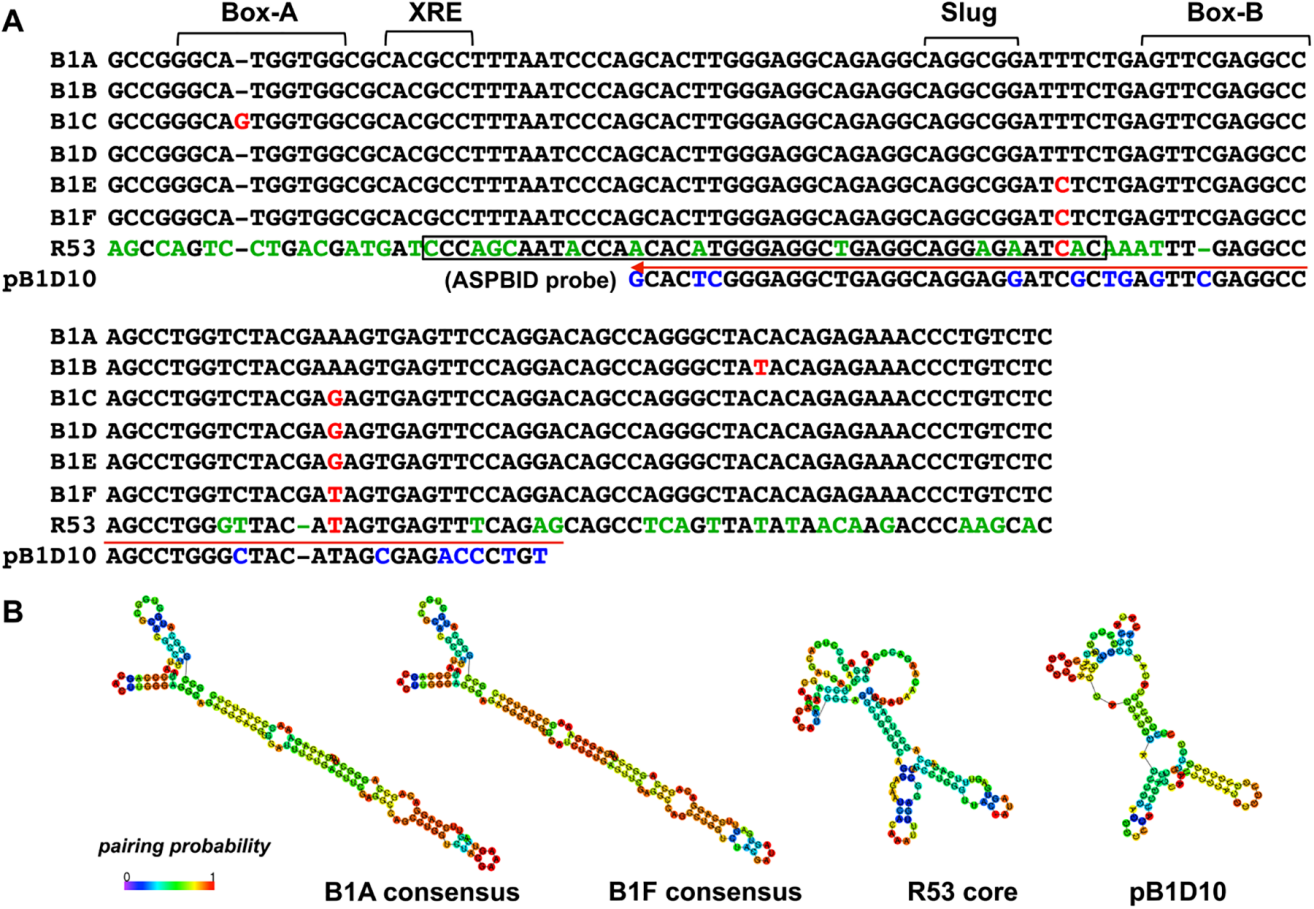
Comparison of the mouse SINE-B1 consensus sequences. **(**A) Consensus sequences (133 bp) of six SINE-B1 subfamilies (A-F) corresponding to the B1 motif of *R53* and the *pB1D10* (pB1D) probe sequence are aligned. The internal promoter sites, box-A and box-B, the xenobiotic response element (XRE) and the epithetial-mesenchymal transition regulator (Slug) TF-binding sites are indicated. The characters colored red indicate the diagnostic positions to distinguish each B1 subfamily and the characters colored green and blue indicate the divergent positions between B1F consensus and *R53* and between *R53* and *pB1D10*, respectively. A red arrow indicates a position of sequences used for the AS2, S2 and pB1D probes. The boxed sequence in *R53* (43 nt) indicates a very high identity (91%) to the corresponding portion of the human ncRNA (LOC105373537, 1.8 kb). (B) Putative secondary structures of the B1A and B1F consensus sequences, and the *R53* RNA core motif (133 nt) and pB1D10 sequences are shown. These structures were predicted using CentroidFold software (http://rtools.cbrc.jp/centroidfold/).

Based on our previous finding that ESCs begin to express the germ cell-specific gene mouse *Vasa* homologue (*Mvh*, also known as *Ddx4)* by 24 hr after co-culture with BMP4-producing M15 cells [23], a repeated PCR-subtraction screening was performed with cDNA that was prepared from cells at 0 hr and 9 hr after the initiation of the co-culture. The noncoding RNA transcript (polyadenylated 587bp) *R53* (GenBank, LC185346) was identified as an early response gene whose expression was initiated in ESCs 9 hr after *Mvh* induction by BMP4 (S1 Fig). The *R53* sequence contains a SINE-B1 motif (133 bp) that belongs to the B1F subfamily, which is one of six subfamilies (B1A-F) that are distinguishable at several diagnostic positions [10, 20] (Fig 1A). B1F sequences are the most highly variable and the most closely related to the *7SL* RNA of ancient origin [10]. In general, retro-transposable SINE elements rely on Pol III transcription that is initiated at the internal promoter, i.e., box-A and box-B, and the 3’-end is composed of an irregular polyA-like sequence [20]. However, because the sequences in the *R53*-B1F motif that correspond to the promoter boxes exhibit a remarkable differences relative to the consensus (Fig 1A) and the motif ends without a polyA-stretch, it seems likely that the *R53*-B1F element itself cannot be transcribed by Pol III and therefore exhibits greatly reduced, if any, transposition activity [24]. Furthermore, the sequence variations of the *R53*-B1F motif revealed a difference in the putative secondary structure, i.e., the *R53*-B1F motif forms a multiple-loop structure similar to that of *pB1D10*, which is a member of the primitive rodent B1 (proto-B1) family [25](Fig 1B); in contrast, other B1 motifs exhibit a single long-stem structure.

**Fig 2:**
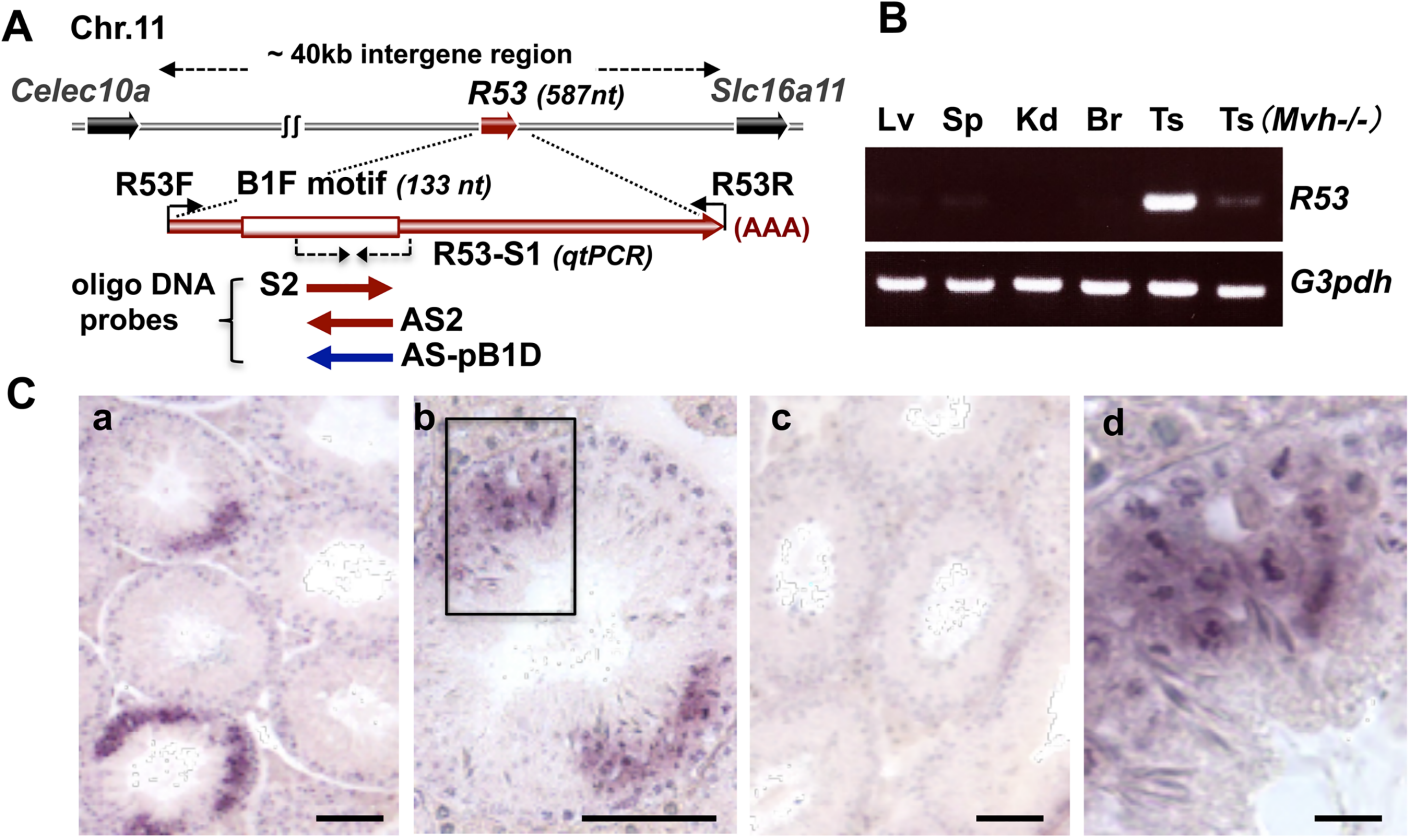
RT-PCR and ISH analyses of *R53* RNA. **(**A) Schematic representation of the *R53* gene locus. The location of Dig-labeled RNA and Dig-oligo DNA probes used for ISH are indicated with arrows, and the region of B1F motif in the *R53* sequence is indicated by the box. (B) The cDNA sequence of *R53* transcript (587 bp) was detected in adult mouse tissues by RT-PCR using R53A (R53F and R53R) as primers (30 cycles) and is shown together with *G3pdh* products (22 cycles) as standards. Single-stranded cDNA was prepared from adult wild-type liver (Lv), spleen (Sp), kidney (Kd), brain (Br) and testis (Ts) tissues, in addition to *Mvh* homozygote (-/-) testis tissue. (C) ISH images of adult testis sections probed with Dig-antisense *R53* RNA (a, b, d) and Dig-sense *R53* RNA (c). (d) High-magnification view of the outlined frame in (b). The scale bars in (a-c) and (d) indicate 100 μm and 20 μm, respectively. Additionally, dotted-line arrows represent the position of R53-S1 primer pair used in Fig 4 and the positions of oligo DNA probes (S2, AS2 and pB1D) used in Fig 4 and S3 Fig are also indicated in the schema.

As illustrated in Fig 2A, the *R53* RNA sequence maps to the intergenic region (approximately 40 kb) between the *Clec10a* (also known as *Mgl1)* and *Slc16a11 (Mct11)* genes (approximately 10kb upstream from *Slc16a11*) on chromosome 11, and the whole sequence of the cloned R53 cDNA (587 bp) matches the corresponding genomic sequence, which indicates that *R53* is an intron-less intergenic lncRNA.

### Expression and localization of *R53* RNA

Among adult tissues, *R53* RNA expression was predominantly detectable in the testis (Fig 2B) and a much lower level of expression was detected in *Mvh* homozygote testis (S2 Fig), in which the spermatogenic process is arrested from the spermatocyte stage onward [26]. These findings are indicative of spermatogenic germ cell-specific expression of *R53* RNA. Indeed, ISH analyses using a digoxigenin (Dig)-labeled antisense RNA probe in adult testis sections revealed a unique finding: *R53* RNA mainly accumulated in a small group of spermatogenic cells in a specific stage of the seminiferous tubules (Fig 2C-a, b), whereas much lower levels expression were detected in other germ cells, such as prophase spermatocytes that reside in the outer and middle layers of seminiferous tubule. A sense RNA probe revealed no positive signal, which indicate that the signals were not due to non-specific binding to other molecules, such as genomic DNA but were rather specific to single-stranded *R53* RNA (Fig 2C-c). Judging from the spermatogenic stage of tubule containing the strongly positive cells and the chromosomal structure, the cells expressing *R53* RNA at the highest level were clearly germ cells in meiotic metaphase, which are found in stage XII during the 12 stages of the cycle (34–38 days per cycle) of the seminiferous epithelium in the mouse [27]. Incidentally, proteins that are specific for the pairing of homologous chromosomes, such as synaptonemal complex proteins (SCPs), are located along the paired meiotic chromosomes, but their accumulation is observed in meiotic prophase spermatocytes. For example, SCP3 (also known as SYCP3) first appears in leptotene spermatocytes and disappears in late meiotic cells [28]. Unlike the *R53* RNA-strongly positive cells, SCP3-positive cells were observed as a multilayer cells inside all the seminiferous tubules (e.g., SCP3-positive spermatocytes as illustrated in Fig 6). To the best of our knowledge, this is the first case in which a molecule has been found to localize predominantly to meiotic metaphase cells in the testis. The stage XII epithelium that contains the meiotic metaphase cells (in both MI and MII stages) has been estimated to account for approximately10% of the whole adult testicular tubules [27]. Moreover, the metaphase-enriched localization of *R53* RNA was also visualized by ISH using Dig-labeled oligo DNA probes (S3 Fig). The antisense 70-mer oligo DNA probe (AS2) corresponding to the R53 B1F element (indicated in Figs 1A and 2A) exhibited the same metaphase-enriched signals as the *R53* antisense RNA probe, whereas the sense probe (S2) that was complementary to AS2 elicited no significant signals. Furthermore, when antisense oligo DNA (ASpB1D, Fig 1A) against a portion of *pB1D10* RNA that exhibits the highest similarity to the AS2 sequence (77.1% identity compared with 72.9% in the case of B1 consensus with AS2) and higher similarity to the B1 consensus (87.1% identity) than the case of AS2 with B1 consensus), was used as a closely related probe for ISH under the same highly stringent condition, ASpBID hybridization signals were strongly detected in most of the spermatogenic cells in the whole seminiferous tubules (S3 Fig), suggesting that metaphase-enriched localization of R53 transcript is not due to cross-hybridization with other B1-family members containing a typical B1 consensus sequence. In addition, the ISH images using both antisense *R53* RNA and oligo DNA probes showed that *R53* transcript was localized in nuclei at the earlier stages (spermatocytes) and in both nuclei and cytosols at the later stages (metaphase and postmeiotic cells).

**Fig 3:**
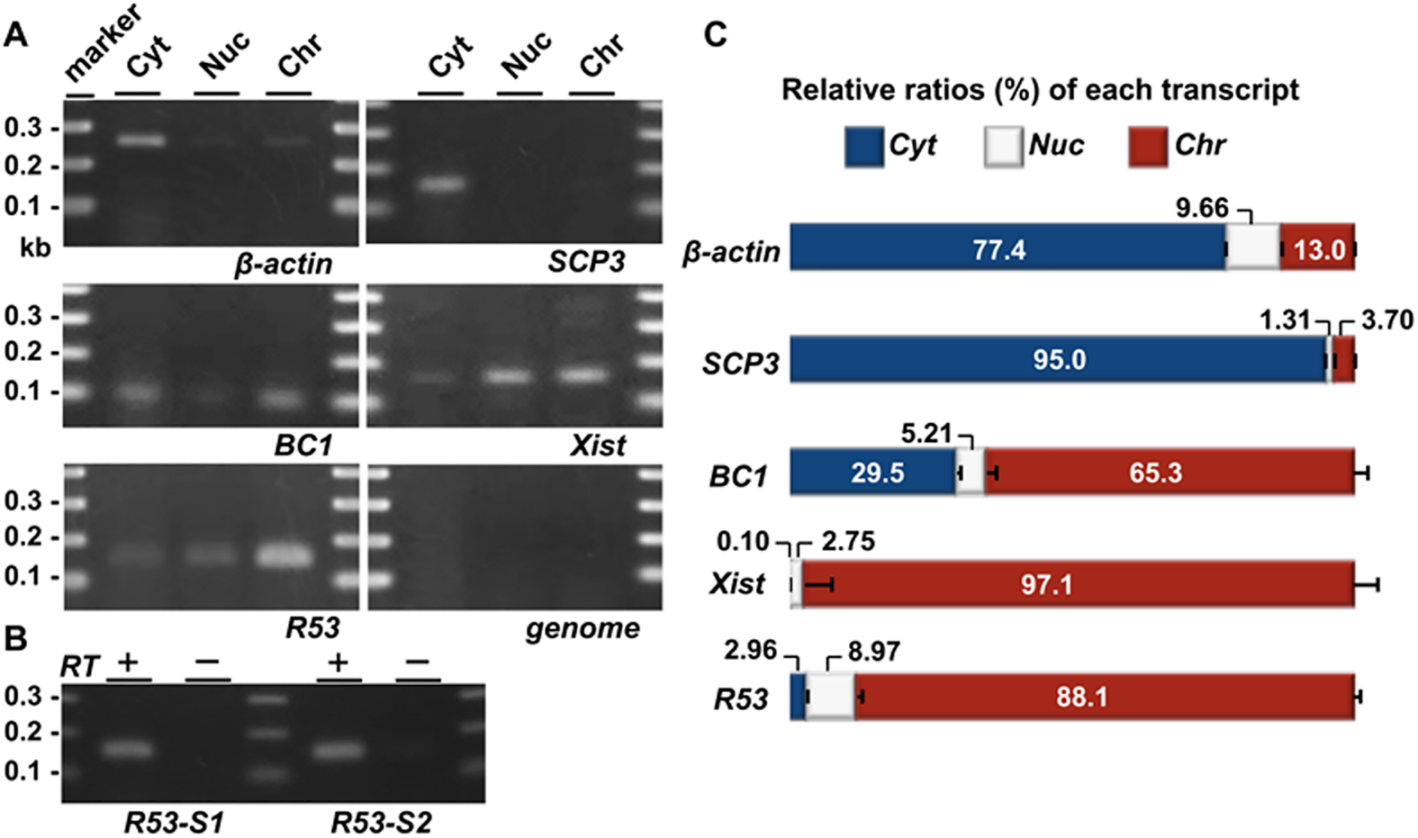
QRT-PCR analyses of the subcellular localization of the RNAs in the testicular cells. Testicular cells were prepared from the testes of mice 22 days after birth; this stage approximately corresponds to the time of the highest level of *R53* expression (Fig 5B). (A) Gel electrophoreses of PCR products using primer pairs for *SCP3* (19 cycles), *BC1* (22 cycles), *Xist* (35 cycles), *β-actin* (18 cycles), *R53* RNA (35 cycles, R53-S2 for the primer detecting a R53-B1F-containing sequence downstream of that of R53-S1) and genotyping of the *Jmjd1C* locus (genome, 35 cycles) in the cytoplasmic extract (Cyt), nuclear soluble (Nuc) and chromatin-bound (Chr) fractions are shown. The genotyping PCR (qJ1C used as the primer) to detect a sequence in the third intron of wild-type *Jmjd1C* gene was performed to demonstrate that there was almost no contamination of the genomic DNA in any fraction. All the primer pairs used are listed in S1 Table. (B) Reverse-transcription was performed using total RNA prepared from the chromatin-bound fraction and oligo-dT primer with (RT+) or without reverse transcriptase (RT-). As in (A), PCR was performed using 2 sets of primers for *R53* RNA (35 cycles) to testify that the PCR products were derived from RT-dependent cDNA. The PCR specificity for *R53* transcript was confirmed by sequencing analyses of the PCR products. (C) The amounts of each transcript in the three subcellular fractions were quantitatively analyzed. The values indicated are the relative levels of each transcript against total values of the three subcellular fractions (set as 100%). The error bars indicate the SEM (n = 6).

Interestingly, the *R53* RNA signals appeared to be tightly associated with metaphase chromatin (Fig 2C-d and S3A-e Fig). The chromatin-associated localization was also detected by reverse transcription (RT) PCR for *R53* RNA in subcellular fractionations of testicular cells (Fig 3A–3C). Among the subcellular fractions for which the accuracy was confirmed by Western blot analysis (S4 Fig), *R53* RNA was found to primarily accumulate in the chromatin-bound fraction in a manner similar to that of *Xist* RNA whereas coding mRNAs, such as *ß-actin* and *Scp3*, were predominantly found in the cytoplasmic fraction. The *BC1* noncoding RNA was detected in the cytoplasm as well as the chromatin-bound fraction, which is consistent with a previous report that observed *BC1* RNA localization in a discrete RNA-protein complex [29]. The *Xist* transcripts that act as a major effector of X-chromosome inactivation are well known to be tightly associated with X-chromosome during spermatogenesis but the antisense *(Tsix)* transcripts are not expressed in the testis [30]. Quantitative reverse transcription (qRT) PCR analyses indicated the amount of *R53* RNA relative to the amount of *ß-actin* mRNA was roughly equal to that of *Xist* RNA and less than 10^−3^-fold that of *BC1* RNA in the testicular total RNA. Additionally, a few *R53* transcripts were also detectable in both the cytoplasm and nuclear extract fractions (Fig 3A), in consistent with the results of ISH.

**Fig 4:**
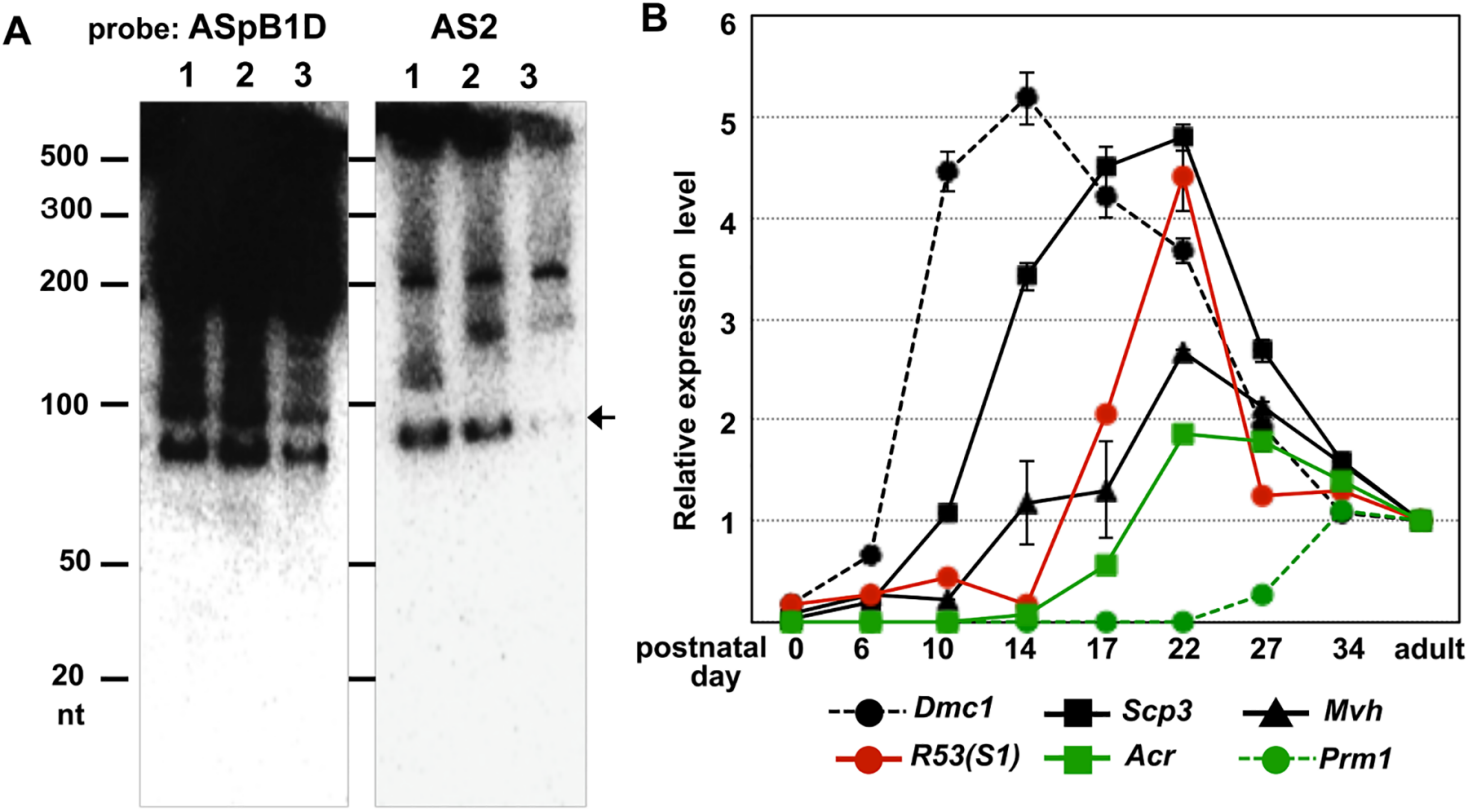
Northern blot analysis and expression kinetics of the *R53* transcript during testicular development. **(**A) A total of 10 μg of total RNA was extracted from each of the adult (4-month-old; lane 1) and immature testes (P14; lane 2) of the wild-type mice and the *Mvh* homozygote adult testis (lane 3) and run using Urea-PAGE. Then, the electro-blotted membranes were hybridized with [^32^P]-labeled ASpB1D probe or AS2 probe. (B) Single-stranded cDNA was prepared from the testes on the indicated days after birth. The expression levels of the *R53* and selected spermatogenic stage-specific genes (including *Dmc1; DNA meiotic recombinase 1* and *Prm1; protamine1*) were quantitatively analyzed with *R53*-S1 and gene-specific primer pairs (listed in S1 Table). The values indicated are the relative values of the expression levels of each gene in the adult (4-month-old) testis (set as 1.0) after standardization to the *β-actin* expression level in the testes. The error bars indicate the SEM (n = 6).

More than 3000 lncRNAs are expressed under developmental control in mouse testis, and many lncRNAs can generate functional small RNAs as precursors [2, 14]. To identify the possible processed RNA product, we performed Northern blot analysis using [^32^P]-labeled oligo DNAs (AS2, S2 and ASpB1D) as probes. As the results, the bands that reacted with the AS2 probe were completely different from those that reacted with ASpBID (Fig 4A and S5 Fig) and S2 probes (S5A Fig). Additionally, the signal intensity in the *Mvh* homozygous (germ cell-poor) testis was dramatically decreased compared with that in wild-type testis (Fig 4A), and there was no hybridized band less than approximately 70 nt; notably, functional small RNAs, such as miRNAs, piRNAs and siRNAs, range in size from approximately 18 to 30 nt. Therefore, it is feasible that the band of approximately 80 nt was the processed product that contained a portion of *R53-*B1F element (overlapping the S2 sequence) derived from the *R53* RNA. Furthermore, a qRT-PCR analysis targeting the *R53*-B1F element using R53-S1 primers (shown in Fig 2) was performed to examine the developmental expression profile during the first wave of spermatogenesis in newborn testis. As shown in Fig 4B, the analysis revealed that low levels of *R53* transcripts became detectable at around postnatal day 10 and the amount was dramatically increased from day 14 to day 22. This indicates that *R53* is initially transcribed in the early prophase (preleptotene or leptotene) and the expression was increased during the late prophase (pachytene and diplotene) stages, and that *R53* RNA expression peaked on approximately day 22 near the timing of emergence of postmeiotic haploid cells. These were consistent with the ISH results, in that detectable signals of *R53* transcripts are found in spermatocytes in most of tubules and the highest accumulation was found in metaphase cells in a few tubules at the stage XII.

### Functional analysis of *R53* RNA in spermatogenesis

Does metaphase chromatin-associated *R53* RNA have any function in spermatogenesis? To answer this question, we used lentivirus-based RNA interference to knock down *R53* expression in the testis. Moreover, we utilized a recently developed organ culture method to complete spermatogenesis from neonatal testes *in vitro* [31] instead of using testes in a living body. This organ culture of testicular tissue has several advantages; e.g., it enables us to continuously trace the developmental changes of the same sample under a microscope and eliminates the difficulty that arises from the individual variation of mice. A lentiviral vector expressing a shRNA targeting *R53* (shR53 lentivirus) was microinjected into the efferent ducts of one of the two testes isolated from a mouse, and a control lentivirus was microinjected into the other testis in the same manner. Next, 4–6 fragments from the treated testes were separately cultivated on an agar block in serum-free medium (S6 Fig). Preliminary experiments using a lentivirus vector expressing *Venus* driven by a *CAG* promoter and wild-type testes (postnatal day 11, P11) as the host revealed that VENUS fluorescence became detectable by day 4 of cultivation in many cells inside the seminiferous tubules, and this fluorescence continued for at least 3 weeks in culture (S7A Fig). Furthermore, the knockdown efficiency of an RNA sequence (a portion of AS2) used to target *R53* expression had been confirmed beforehand using a cultured cell line carrying an *R53* RNA (total-length of the R53 cDNA) forced-expression vector (S7B Fig). The *R53* RNA expression was efficiently reduced to below 20% that of the control by the *R53* shRNA-treatment.

**Fig 5:**
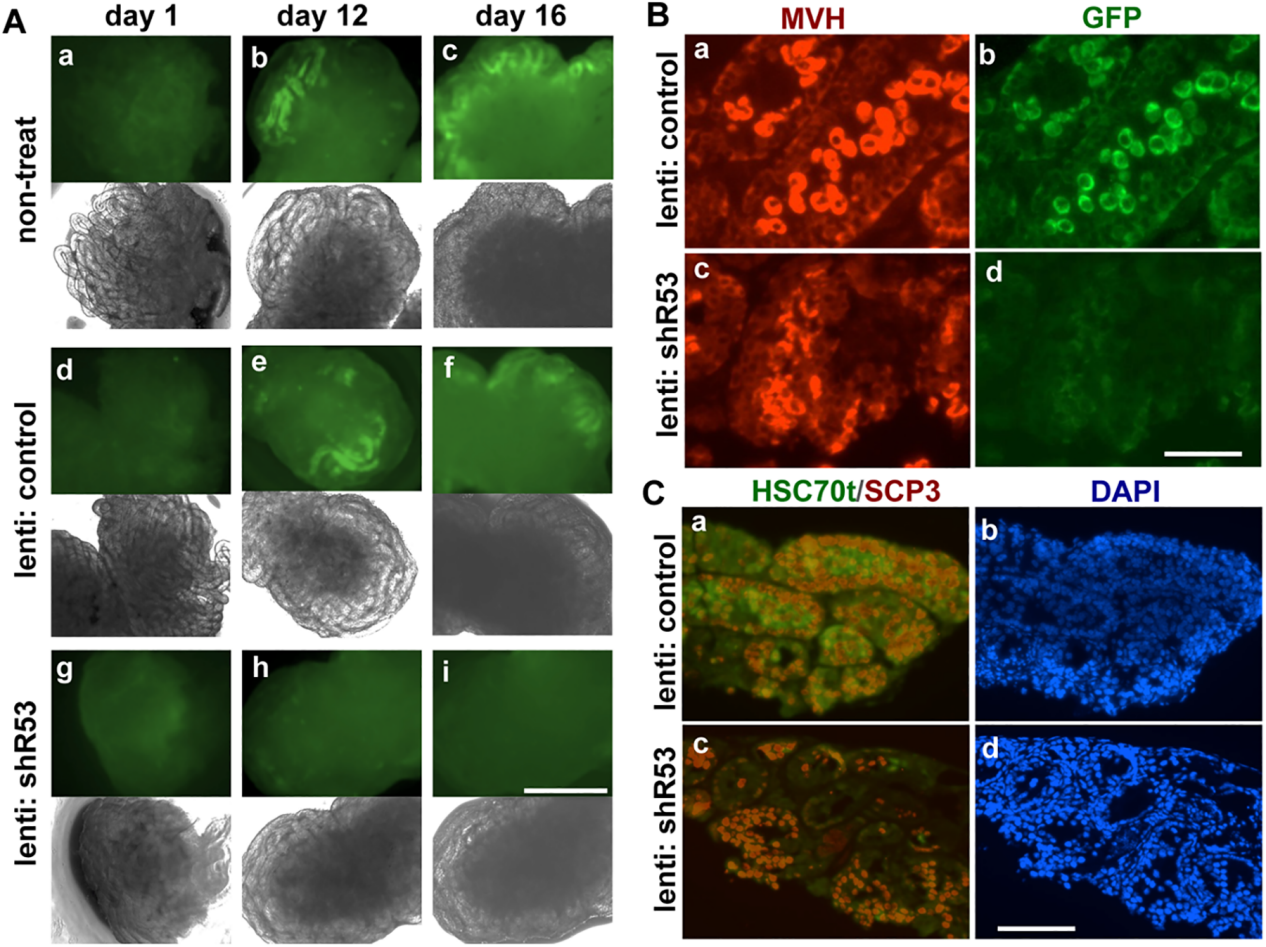
The appearance of *Acrosin-GFP*-positive cells in organ culture. **(**A) Fragments of immature testes (P11) from *Acr-GFP* transgenic littermates were cultivated on agarose blocks. Non-treated (a-c), control lentivirus-injected (d-f) and shR53 lentivirus-injected (g-i) fragments were observed under a fluorescence microscope on day 1 (a, d, g), day 12 (b, e, h) and day 16 (c, f, i) of culture. The photographs beneath (a)-(i) are phase contrast images of each fluorescent field. The scale bar in (i) for (a-i) is 1 mm. (B) Sections from a control lentivirus-injected fragment (a, b) and a shR53 lentivirus-injected (c, d) fragment after 16 days of culture were double-stained with anti-MVH (red) and anti-GFP (green). The scale bar in (d) for (a-d) is 50 μm. (C) Sections from a control lentivirus-injected fragment (a, b) and a shR53 lentivirus-injected (c, d) fragment after 16 days of culture were double-stained with anti-HSC70t (green) and anti-SCP3 (red). The merged images (a, c) are shown together with nuclear DAPI staining (b, d). The scale bar in (d) for (a-d) is 100 μm.

First, we utilized the immature testes of littermates of transgenic male mice (P11) that carried an *Acrosin (Acr)-GFP* gene as the host testes [31]. Herein, differentiation of *Acr*-expressing spermatids could be visualized as the appearance of GFP-positive cells. On the first day of culture, no GFP-positive cells were observed (Fig 5A), and immunohistochemical analysis of a P12 testis section also demonstrated the absence of spermatids stained specifically for anti-HSC70t (heat shock cognate 70 kDa protein in the testes). Spermatocytes stained positively for anti-SCP3 began to appear at approximately P12 (S7C Fig). After 12 and 16 days of culture, *Acr*-GFP-positive cells were observed in both the non-treated and control lentivirus-injected testis fragments, respectively (Fig 5A). Typically, the *Acr-*GFP expression became more clearly detectable earlier in the tubules at the periphery than in those in the center region of the testis fragments. In contrast, although spermatogenic cells that stained positive for anti-MVH clearly existed, few *Acr-*GFP positive cells appeared in the shR53 lentivirus-injected fragments (Fig 5A and 5B). This result was not due to a delay in appearance because no additional GFP was observed even after prolonged culture (1 additional week; data not shown). Immunohistochemical analyses of the fragments (16 days of culture) revealed that few spermatids stained with anti-HSC70t in the shR53 lentivirus-injected fragments. This result contrasts the case of the control lentivirus-injected testes in which multilayered HSC70t-positive spermatids were found inside the SCP3-positive spermatocytes (Fig 5C), which suggests that *R53* knockdown elicited an inhibitory effect on the appearance of postmeiotic germ cells.

**Fig 6:**
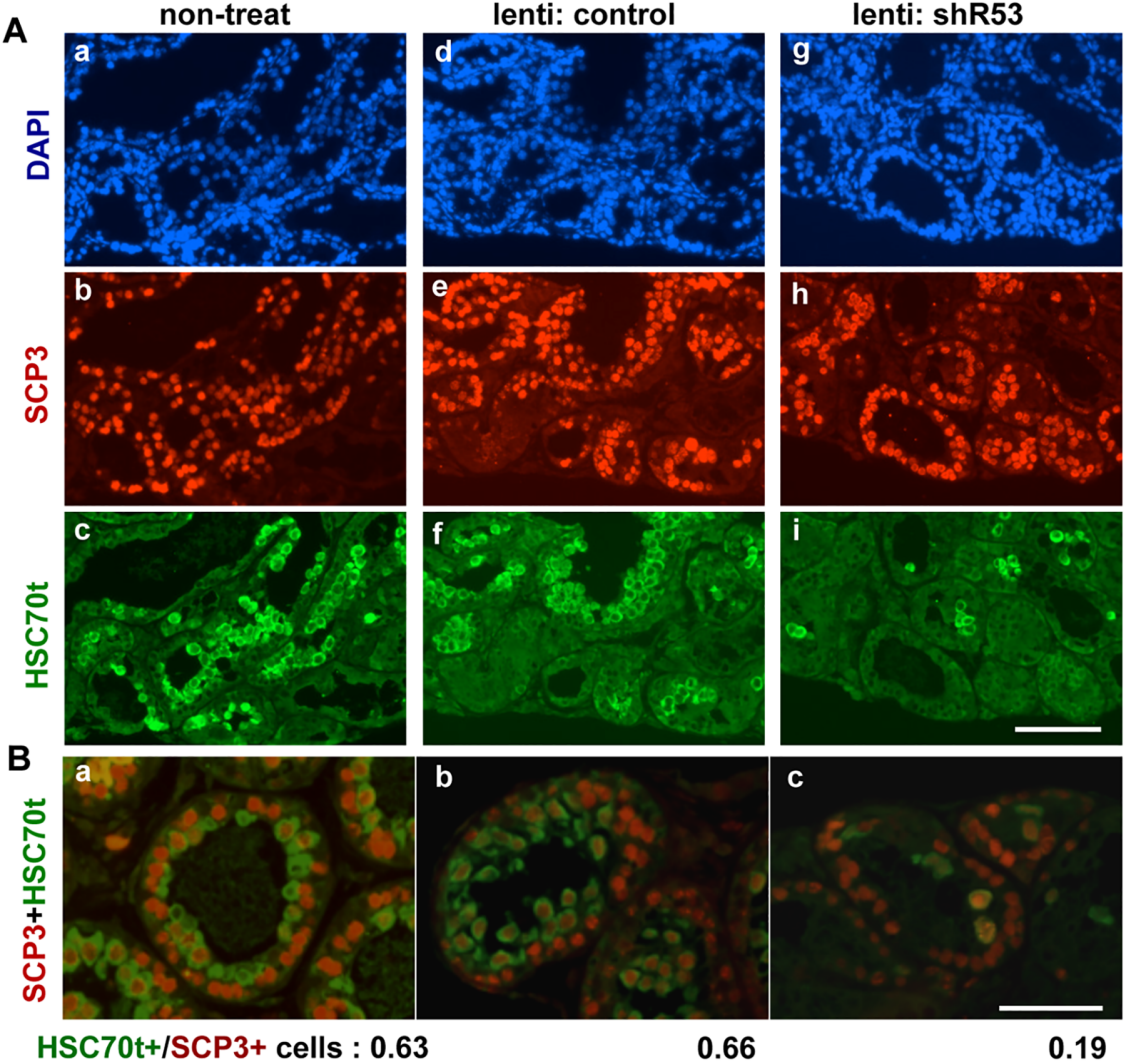
Immunostainings of the organ-cultured fragments. **(**A) Fragments of immature testes (P11) from wild-type littermates were cultivated for 21 days. Histological sections from non-treated (a-c), control lentivirus-injected (d-f), shR53 lentivirus-injected (g-i) fragments were stained with DAPI (a, d, g), anti-SCP3 (b, e, h) and anti-HSC70t (c, f, i). The scale bar in (i) for (a)-(i) is 200 μm. (B) Double-stainined anti-SCP3 (red) and anti-HSC70t (green) images from non-treated (a), control lentivirus-injected (b) and shR53 lentivirus-injected (c) fragments. The scale bar in (c) for (a-c) is 100 μm. The ratios of HSC70t-positive cells to SCP3-positive cells (indicated below the photographs) were roughly estimated by counting the number of positively stained cells in a whole field for each of 6 sections (the numbers of SCP3-positive cells that were counted were 3000–4000 cells in each case, n = 3).

**Fig 7:**
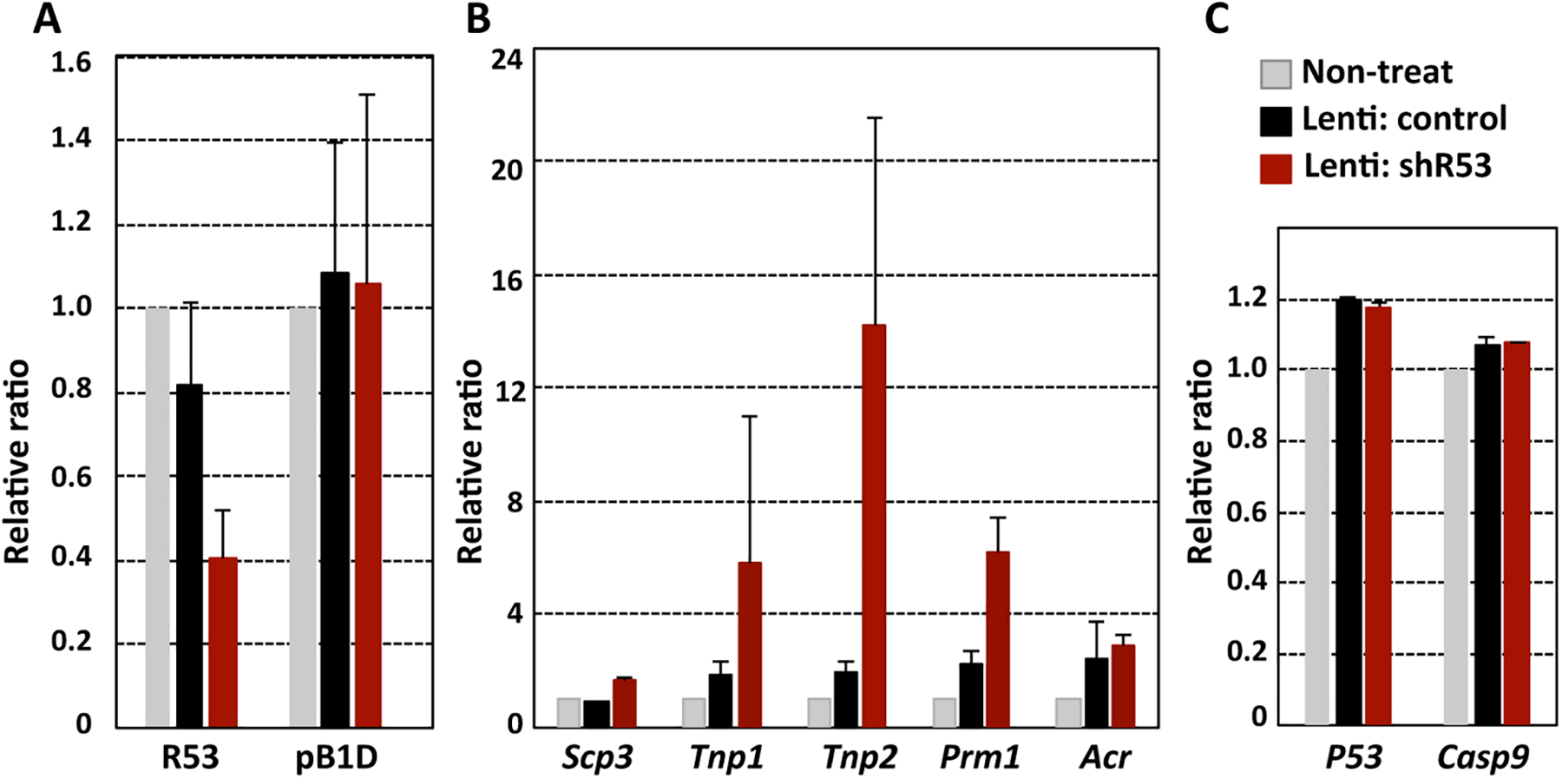
Changes of expression levels of *R53* and selected genes due to *R53* knockdown. Single-stranded cDNA was prepared from the testicular fragments (P11) on day 4 after the initiation of organ culture. (A) The expression levels of *R53* RNA in non-treated, control lentivirus-injected and shR53 lentivirus-injected fragments were quantitatively analyzed with R53-S1primer. Similarly, the expression of *pB1D*, which is the most closely related transcript, was analyzed to look for off-target effects. The values indicated are the relative values of the expression level of each gene for the non-treated fragment (set as 1.0) after standardization to the *Mvh* (germ cell-specific gene) expression level for each fragment. The error bars indicate the SEM (n = 6). (B) and (C) As in (A), the values indicated are the relative values of the expression level of each gene in the non-treated fragment (set as 1.0) after standardization to the *Mvh* expression level in each fragments. The error bars indicate the SEM (n = 6–8).

Second, quantitative analyses of the shR53 knockdown effect were performed using immature testes (P9-P12) of the littermates of the wild-type (ICR) mice. Immunohistochemical staining of the testicular fragments after 21 days of culture reproducibly demonstrated that the numbers of HSC70t-positive spermatids were remarkably decreased in the shR53 lentivirus-injected testis fragments (Fig 6A and S8 Fig). Based on double-stained images using antibodies against HSC70t and SCP3 such as those presented in Fig 6B, the ratios of the numbers of HSC70t-positive spermatids to SCP3-positive spermatocytes were estimated, and the scores were 0.63 (± 0.03: SEM), 0.66 (± 0.03) and 0.19 (± 0.11) in the non-treated, control lentivirus-injected and shR53 lentivirus-injected testicular fragments, respectively, which suggests specific decrease in the number of postmeiotic germ cells in the shR53-treated testicular fragments. Moreover, qRT-PCR analyses were performed using total RNAs that were prepared from each testicular fragment (P11) after 4 days of organ culture, which approximately corresponded to *in vivo* testis on neonatal day 15 (P15). At the point, the 1^st^ wave spermatogenesis has proceeded to the meiotic prophase, and the expression of *R53* RNA has commenced (Fig 4B). First, the results demonstrated that *R53* RNA expression was reduced to 30–40% in the shR53 lentivirus-injection sample compared with the non-treated sample, whereas the expression of *pB1D* RNA (the RNA that exhibits the highest sequence homology with *R53* RNA) was not affected (Fig 7A). Furthermore, to examine the developmental process in each testicular fragment, the expression of several meiotic and postmeiotic marker genes was analyzed. Surprisingly, the results displayed in Fig 7B revealed that the expression levels of *Prm1*, *Tnp (transition protein) 1 and Tnp2* were dramatically increased by the *R53* knockdown treatment, although the expression of premeiotic genes, such as *Mvh* and *Scp3*, was not affected. These results suggest the possibility that *R53* lncRNA plays a crucial role in chromatin-mediated gene regulation for a subset of spermatid genes involved in chromatin remodeling during meiotic prophase in spermatogenesis.

## Discussion

### *R53* RNA accumulates on meiotic metaphase chromatin

Our present study demonstrated the existence of a lncRNA containing a SINE-B1F motif termed *R53* RNA that predominantly localizes to meiotic metaphase chromatin during spermatogenesis. The findings of this study also revealed that *R53* RNA plays an indispensable role in meiotic progression because the knockdown of *R53* expression hampered progression to postmeiotic stages and was accompanied by the deregulation of some spermiogenesis-related genes.

Currently, there is growing interest in analyses that indicate that many lncRNAs have structural or functional roles in chromosome [32, 33]. *In situ* visualization of RNA using concentration x time (Cot)-1 highly repeated DNA probes have demonstrated that abundant RNA composed predominantly of repeat sequence, including LINE-1-derived RNA, are main component that are associated with the euchromatin region of the interphase chromosome [17]. These interphase chromosome-associated RNAs (iCARs) are stable during interphase but detach from the prophase chromosome during condensation prior to metaphase [17]. The iCARs include approximately 400 ncRNA species, and their roles are assumed to be related to the maintenance of de-condensed chromosomal state, and this maintenance is probably mediated by the recruitment of chromosome-associated RBPs that also possess histone modifying activities that lead to chromatin remodeling, such as HP1, MOF and polycomb group proteins [9, 12].

A recent investigation of metaphase CARs (mCARs) using mouse 3T3 cells cataloged over 1000 species of RNAs that are predominantly composed of ncRNAs [22] in which SINE-derived ncRNA are an integral component. LINE-RNAs are enriched in iCARs, which indicates a massive redistribution of CARs during the mitotic cell cycle. Furthermore, proteome analysis has revealed hundreds of RBPs that are associated with the mitotic chromatin [34]. These results imply the possibility that SINE-derived mCAR are involved in repressing gene expression in condensed chromatin because they act as scaffold elements for the cooperative binding of chromatin-associated complexes in a cell-cycle dependent manner, whereas LINE-derived iCARs are involved in the formation of transcription-favorable chromosome structures. Despite these findings, no investigation has focused on meiotic mCARs in mammals; therefore, *R53* is the first lncRNA to be identified as a component of the meiotic metaphase chromatin.

Sun *et al*. [2] revealed that 3025 species of lncRNAs are differentially expressed during testicular development (1062 and 1963 lncRNAs were significantly down- and up-regulated in adulthood, respectively). Moreover, these authors found that some of their loci overlap (exonic or intronic lncRNAs) or are adjacent (intergenic lncRNAs) to key spermatogenesis-regulating genes, such as *Plzf*, *Kit*, *Tnp1 and Prm1*, which led to the interesting hypothesis that some lncRNAs are coordinately transcribed with their associated genes that encode spermatogenesis-regulating proteins and play a critical role in the transcriptional regulation of these genes. In contrast, the functions of only a limited number of testicular lncRNAs have been explored. *Testis specific X-linked (Tsx)*, which is located within the X-inactivation center, is expressed in meiotic germ cells, and *Tsx*-deficiency is associated with pachytene-specific apoptosis [35]. *Meiotic recombination hot spot locus (Mrhl*) RNA is a nuclear enriched lncRNA that negatively regulates Wnt-signaling together with its partner DDX5, which is a DEAD-box RNA helicase, in a cell line that was immortalized from spermatogonia [36]. Interestingly, the primary transcript of the *Mrhl* RNA is known to be processed into an approximately 80 nt intermediate RNA by the Drosha machinery [37]. Another example is *BC1* lncRNA, which contains a SINE-ID motif and is transcribed at high levels in premeiotic germ cells ranging from spermatogonia to spermatocytes in the testis as well as in neurons in the brain [18]. Incidentally, dendritic *BC1* RNA functions in translational repression at the initiation level by binding to eIF4A, which is a DEAD-box RNA helicase [19]. However, chromosome-associated lncRNAs that act in spermatogenesis have not been reported to date.

### What specific role does *R53* RNA play in meiotic chromatin?

A clear difference between meiotic chromosomes and mitotic chromosomes is the pairing of two homologous chromosomes that is mediated by synaptonemal complexes (SCs) and followed by the successive formation of chromatin cross-overs, i.e., chiasmata. Interestingly, chromatin immunoprecipitation using anti-SCP3 has revealed that SC-associated DNA is predominantly composed of repeated sequences, such as SINEs and LINEs (20% and 39% of the total, respectively) [38]. Furthermore, a meiosis-specific lncRNA *(Sme2)* has been found to play an essential role in homologous chromosome pairing in yeast [39]. However, these meiosis-specific events occur during prophase of the first meiotic division prior to *R53* RNA accumulation on the metaphase chromatin. Apparently, in contrast to the metaphase chromatin, faint ISH signals were detected in the SCP-positive prophase spermatocytes with *R53* probes (Fig 2C and S3 Fig), which suggests that it is unlikely that *R53* RNA directly contributes to the assembly of SCs. Rather, it may be more likely that *R53* RNA plays a role in releasing the homologous chromatin from the pairing machinery in a metaphase-specific manner. If this is the case, it would be reasonable to suggest that a segregation defect causes the remarkable decrease in postmeiotic cells that was observed in the *R53* knockdown testes.

Another possibility is that *R53* RNA is involved in the transcriptional regulation of key spermatogenesis genes. This idea is supported by the finding that *R53* knockdown led to the precocious up-regulation of several postmeiosis genes (Fig 7B). In this case, *R53* could be thought to function as a transcriptional repressor of a subset of spermiogenesis genes in meiotic prophase chromosomes. As illustrated in Fig 2C, Fig 4B and S3 Fig, ISH and developmental kinetic analyses demonstrated the predominant expression of *R53* transcripts in metaphase cells, but *R53* expression was not exclusively specific to metaphase cells. The onset of *R53* RNA expression was detected during prophase prior to metaphase stage (Fig 4B). Moreover, weak but distinct ISH signals with AS2 probe were also detected on the chromosomes of prophase spermatocytes and in the cytosol of metaphase and postmeiotic cells (S3 Fig). Incidentally, similar localization change of ncRNA transcripts during meiosis was recently reported in a testis-specific lncRNA-HSVIII [40]. Therefore, it is possible to speculate that *R53* RNA initiates the association with condensing chromosomes during meiotic prophase, the rapid accumulation persists into metaphase chromatins, and the initial function during meiotic prophase is transcriptional repression that is probably via interaction with chromatin-associated RBPs; this process would be similar to the putative function of SINE-derived mCARs. In either case, the drastic reduction of postmeiotic cells upon *R53* knockdown appeared to be the result of abnormal meiotic progression and not the direct induction of apoptotic cell death because typical apoptosis-specific genes (*P53* and *Casperse 9*) were not immediately activated by the *R53* knockdown (Fig 7C).

The B1F motif of *R53* RNA belongs to the SINE-B1/*Alu*-family, which is shared between the rodent and primate genomes. In contrast, no *Alu* element with high homology with the sequence of *R53* cDNA is present in the human genome, whereas sequences that are highly homologous with only a portion of *R53* sequence (e.g., the 43 nt sequence presented in Fig 1) are found in humans. This finding is due to the sequence diversity of SINE repeat elements in general. However, the absence of homologs with a conserved sequence does not necessarily negate the existence of lncRNAs that exhibit functional similarities to *R53* RNA in other animal species. Although *Xist* RNAs are known to exhibit little sequence conservation between humans and mice, they have a common function [41]. As described above, lncRNAs in CARs are thought to perform their functions via binding their partner RBPs as scaffolds that recruit other chromatin-associated proteins, such as epigenetic modifiers [2, 9, 12]. Furthermore, a detailed investigation of the RBPs that bind to *BC1* RNA suggested that the secondary structure of *BC1* RNA comprises the SINE-ID motif is important for the binding specificity [29]. Accordingly, if a partner RBP recognizes specific nucleotides at specified positions and the surrounding secondary structure of the accompanying ncRNA molecule, it is possible that, regardless of the sequence identity with *R53*, there are meiotic CARs with function similar to that of *R53* in other animal species.

Further analysis is needed to clarify the partner RBPs of *R53* RNA during spermatogenesis. Thus far, many germline cell- or spermatogenesis-specific RBPs that are enriched in the germplasm (*nuage*) have been identified, including DEAD-box and Tudor-domain proteins [9], but chromosome-associated RBPs that function in spermatogenesis have not been explored in mammals. Further investigation will be required to elucidate the mechanism by which mCARs regulate the dynamic state of meiotic chromatin during spermatogenesis.

## Materials and methods

### Animals

*Mvh* homozygous mice were generated by intercrossing heterozygotes as previously described [26]. *Acr-GFP* homozygous transgenic mice (originally provided by RIKEN BRC through the National Bio-Resource Project of MEXT, Japan) and Jcl:ICR wild-type mice were bred by intercrossing, and the male littermate (total 36) pups on postnatal 9–12 days were used for the culture experiments. Jcl:ICR male (total approximately 80) mice were used for ISH, immunoblot, RNA extraction and histochemical analyses in this study. All animal experiments conformed to the Guide for Care and Use of Laboratory Animals and were fully approved by the Keio University Institutional Animal Care and Use Committee. All animal care and experimental procedures were performed in accordance with the institutional Committee of Laboratory Animals and the Institutional Guidelines on Animal Experimentation at Keio University and Yokohama City University.

### In situ hybridization

All ISH procedures were performed essentially according to the previously described methods [42]. Briefly, paraffin-embedded sections (10 μm) of 4% paraformaldehyde-fixed testes were used for hybridization with Dig-labeled probes. The sense/antisense RNA probes were prepared using DIG RNA labeling kit SP6/T7 (Roche) and plasmid DNA containing *R53* cDNA /pGEM-T Easy as the template. Synthetic oligo DNA (the probe sequences are listed in S1 Table) was labeled using a DIG oligonucleotide 3’-End Labeling Kit (Roche) according to the manufacturer’s instructions. ISH with the Dig-labeled probes was performed in the following relatively high-stringent conditions, i.e., hybridization in a solution (50% formamide, 10% dextran sulfate, 1xDenhardt’s solution, 300 mM NaCl, 10mM DTT, 20 mM sodium acetate, 5 mM EDTA, 0.1 mg/ml yeast tRNA) at 42℃, overnight followed by washing in 50% formamide, 2xSSC, 100mM DTT at 65℃ for 30 min and finally in 0.1xSSC at 50℃ for 4 hr. The detection of the ISH signals was performed using a DIG Nucleic Acid Detection Kit (Roche) according to the manufacturer’s manual.

### Northern blot

Total RNA was extracted from adult testes (wild-type and *Mvh* homozygote) and organ cultured tissues (4-day culture) using Sepasol-RNAI SuperG (Nacalai Tesque) according to the manufacturer’s manual. A total of 20 *μ*g of total RNA was electrophoresed on a 10% Novex^TM^ TBE-Urea Gel (Invitrogen) and transferred to a Hybond-N+ membrane (Amersham Biosciences) using semi-dry electroblotter at 400mA for 30 min and subsequently UV crosslinked. Oligo DNA probes (10 ng each) were labeled using gamma [^32^P]-ATP with a MEGALABEL Kit (Takara-Shuzo). Hybridization was performed in a hybridization buffer (10% SDS, 10% dextran sulfate, 1M NaCl, 0.5mg/ml sonicated salmon sperm DNA) at 65℃ overnight. The membranes were then washed twice in 2xSSC and 0.5% SDS at room temperature for 30min and in 0.2xSSC and 0.5% SDS at 65℃ for 30 min. Next, the signals were visualized using a BAS-3000 image-analyzer (GE-HealthCare).

### RT-PCR and qRT-PCR

Total RNA was extracted from mouse tissues using a Qiagen RNeasy Kit (Qiagen) or Sepasol-RNAI SuperG (Nacalai Tesque) and was DNase-treated (Promega RQ1 RNase-free DNase). Single-stranded cDNA was prepared from the total RNA using an oligo-dT primer and a Superscript III First-Strand cDNA Synthesis Kit (Invitrogen) in accordance with the manufacturers’ instructions. The RT (reverse transcription)-PCR was performed with Ex-Taq (Takara-Shuzo), and the qRT-PCR results were analyzed using a SYBR Premix EX TaqII Kit (Takara) and ViiA7 (Applied Biosystems). The qRT-PCR reactions were normalized against the *β-actin* transcript levels. The primer pairs used for the cDNA analyses are listed in S1 Table.

### RNA and protein extraction from subcellular fraction

The subcellular fractions of cytoplasmic, nuclear-soluble and chromatin-bound extracts were prepared from 1x10^7^ P22 mouse testes cells using a Subcellular Protein Fractionation Kit for Tissues (Thermo Scientific) in accordance with the manufacturers’ instructions with exception that RNasin Ribonuclease Inhibitors (40 U/ml, Promega) were added to all of the buffers used for this fractionation. The total RNA from each subcellular extract was prepared via the addition of at least 4x volume of Sepasol-RNAI SuperG and suspended in 20 μl of H_2_O after DNase treatment. Single-stranded cDNA (final volume 20 μl) was prepared using 4 μl of the total RNA solution from each fraction as the template, and 0.5 μl of cDNA per reaction was analyzed by qRT-PCR (the primers are listed in S1 Table). For the immune-blot analysis, 1/50 of the volume of the initial cell suspension and each subcellular fraction were prepared via the addition of a 1/3 volume of 4x Laemmli sample buffer (BioRad).

### Knockdown by lentivirus-mediated shRNA

The *R53* RNA-sequence specific shDNA used in the experiment was 5’-AAACTCACTATGTAACCCAGG-3’, i.e., a portion of the AS2 sequence, which was cloned into the pLKO.1-CMV-tGFP packaging plasmid (Mission RNAi, Sigma-Aldrich). The plasmid DNAs and the solutions for the shR53 lentivirus (custom-made) and control lentivirus (non-Target shRNA control, SHC016) were purchased from Sigma-Aldrich. A lentivirus vector carrying a *Venus* cDNA driven by *CAG* promoter, i.e., pCS-CA-Venus (provided by RIKENBRC DNA Bank, http://cfm.brc.riken.jp/), was transfected with the other three plasmids (pGag/Pol, pRev, pVSV-G) into HEK293TFK cells to produce lentivirus particles. The microinjections of lentivirus solution (approximately 10 μl of 10^6^ TU/ml per testis) into the efferent ducts of the testis of the littermate pups (P9-12) were performed essentially according to the detailed protocol described by Sato *et al*. [43]. After lentivirus injection, the testes were fragmented to 4–6 pieces, and the fragments were then positioned on agarose blocks soaked in organ culture medium (S5 Fig).

### Testis organ culture

The organ cultures of the testis fragments used in this study were created according to a previously described method [31] with a few modifications. The culture medium was 10% KnockOut serum replacement (KSR; Gibco)/ alpha-Minimum Essential Medium (MEM; Gibco) supplemented with a MEM non-essential amino acid solution (x1, Gibco), 1mM sodium pyruvate (Gibco) and a penicillin/streptomycin/glutamine solution (x1, Gibco). The fragmented testis tissues were positioned on agarose blocks that were in a 12-well culture plate with the culture medium. The agarose blocks were constructed as follows; 1.5% (W/V) Agarose LO3 (Takara-Shuzo) was solidified in a 48-well culture plate, and the cylindrical agarose gels were pulled out and pre-equilibrated in the culture medium for more than 24 hrs.

### Immunohistochemical staining

The testes and organ-cultured tissues were fixed in 4% paraformaldehyde in PBS and embedded in paraffin. After deparaffinization, 5-μm-thick sections were treated with Target Retrieval Solution (Dako, S1699) for unmasking, blocked with 3% BSA/0.1% Tween 20 in PBS and then stained with antibodies diluted in 1% BSA/0.1% Tween 20 in PBS. The primary antibodies, including anti-HSC70t [44] and anti-MVH [45], and the secondary antibodies used in this study are listed in S2 Table. Acr-GFP and fluorescent immunohistochemical images were observed with an Olympus IX71 inverted microscope and acquired with MetaMorph software.

## Supporting information

S1 Fig Isolation of *R53* RNA by repeated PCR subtraction. To isolate early response genes that were activated following germ cell induction from ESCs, repeated PCR subtraction was performed using cDNA prepared from a mixture of ESCs and BMP4-producing M15 cells versus cDNA from a suspension culture of mixed cells for 9hrs. RT-PCR using R53A (R53F and R53R) as primers to produce a *R53* cDNA together with *G3pdh* products, which were used as a standard, revealed that *R53* RNA expression became detectable 9 hr after the initiation of co-culture.
(TIF)

S2 Fig QRT-PCR for *R53* expression. Single-stranded cDNA was prepared from adult testes (4-month-old) of *Mvh* heterozygote (+/-) and homozygote (-/-) mice. *R53* expression was quantitatively analyzed with 2 pairs of primers, i.e., R53A (which detected the R53 cDNA, 587bp) and R53-S1 (which detected a R53-B1F element). The values are given as the relative ratios against the *G3pdh* gene expression (x10^-4^) and are average of three independent experiments.
(TIF)

S3 Fig ISH analyses of *R53* RNA using Dig-labeled oligo DNA probes. ISH against adult testis sections was performed using Dig-labeled 70-mer oligo DNAs, AS2 (antisense), S2 (sense) and ASpB1D (antisense) as probes (the positions are represented in Fig 2 and the sequences are listed in S1 Table and Fig 1). Hybridization and washing were performed in the same stringent condition and the colorization with an alkaline phosphatase reaction was performed at the same time for all the probes. A) ISH images of colorization for 4 hrs. (a) S2, (b) AS2 and (c) pB1D were used for probes, respectively. (d) Hybridization without probe that shows a background staining by AP- conjugated anti-Dig antibody. (e) High-magnification view of a portion of (b) showing the positive signals localized on the metaphase chromatin. The scale bar in (a) for (a—d) is 200 μm, and the bar in (e) is 20 μm. B) ISH images of clorization for approximately 16 hrs to strengthen the signals. (a) S2, (b) AS2 and (c) pB1D were used as probes. (d) and (e) High-magnification views of two outlined frames in (b). (d) and (e) are tubules at stage XII and stage V, respectively. Arrows in (c) indicate late pachytene spermatocytes. Compared with a staining image using sense probe in (a), the positive signals of *R53* RNA become to be detectable on the pairing chromosomes of prophase spermatocytes and in the cytosol/nuclei of round spermatids. The scale bar in (a) for (a—c) and in (d) for (d, e) is 200 μm and 50 μm, respectively.
(TIF)

S4 Fig Immunoblot analysis of subcellular protein fractionation. SDS-sample solutions were prepared from whole-cell extract and subcellular fractions (Cyt, Nuc and Chr) and a half volume of each solution was subjected to SDS-PAGE. The blotted membrane was reacted with a mixture of anti-HSC70t and anti-Histone H3 and visualized via a HRP reaction using anti-rabbit IgG secondary antibody. The antibodies used in this analysis are listed in S2 Table.
(TIF)

S5 Fig Northern blot analyses using *R53* oligo DNA probes. (A) A total of 10 μg of total RNA extracted from adult (4-month-old) testes was used for PAGE (duplicated in two lanes), and then two sets of electro-blotted membranes were hybridized with AS2 antisense or S2 sense probes. The arrow indicates the band of approximately 80 nt specifically hybridized with AS2 probe. (B) A total of 40 μg (lane 1) and 20 μg (lane 2) of total RNA prepared from adult testis (3-month-old) were electrophoresed using a 1.1% formalin-agarose gel. The blotted membrane was hybridized with AS2 antisense probe and then the hybridizing signals were detected as described in materials and methods.
(TIF)

S6 Fig Flow chart of our knockdown procedure. After intratubular injection of the lentivirus particles, the testes were fragmented and then positioned on the agarose blocks soaked in organ culture medium.
(TIF)

S7 Fig Preliminary results from shR53 knockdown experiment. (A) The efficiency of lentivirus-mediated gene transduction into testicular tubules was examined with a lentivirus vector carrying a *CAG-Venus* gene. Non-treated and lentivirus-injected testes (from wild-type littermates at P11) were fragmented into 4 pieces and cultivated on agarose blocks. Four days later, non-treated (a, b) and lentivirus-injected (c, d) fragments were examined their Venus expression as shown in each phase contrast image. (e, f) Pictures of a 21 day culture of a lentivirus-injected fragment. The scale bar in (a) is for (a-d), and the scale bar in (e) is for (e, f), the bars are100 μm and 1mm, respectively. (B) The knockdown efficiency of shR53 RNA was first examined using a HEK293T cell line carrying a *R53* forced-expression vector. Three days after transfection with the control lentivirus or the shR53 lentivirus vector DNAs, the single-stranded cDNAs were prepared from these transfected cells and non-transfected cells (without DNA) were used as the control. The *R53* expression was quantitatively analyzed with two different primer pairs, i.e., for R53S1and R53S2, to detect the transcripts that contained an R53-B1F element. The values indicated are the relative values of the *R53* expression level of the non-treated cells (set as 1.0) after standardization to the *β-actin* expression level. The error bars indicate the SEM (n = 6). (C) Sections of a P12 testis were double-stained with anti-HSC70t (a) and anti-SCP3 (b); Alexa Fluor-488 labeled anti-rabbit IgG and Alexa Fluor-568 labeled anti-mouse IgG were used as the secondary antibodies, respectively. The views of phase-contrast and nuclear staining with DAPI are shown in (c) and (d). The scale bar in (d) is 100 μm.
(TIF)

S8 Fig Immunostainings of organ-cultured fragments. Fragments of immature testes (P9) from wild-type littermates were cultivated for 21 days. Histological sections from non-treated (a-d), control lentivirus-injected (e-h) and shR53 lentivirus-injected (i-l) fragments were stained with anti-SCP3 (a, e, i), anti-HSC70t (b, f, j) and DAPI (c, g, k), and the results are shown together with each phase contrast image (d, h, l). The scale bar in (l) for (a)-(l) is 100 μm.
(TIF)

S1 Protocol Supplementary methods and materials. (DOCX)

S1 Table Sequences of primers and probes used in this study. (DOCX)

S2 Table Antibodies used in this study. (DOCX)
