# Supplementary figures and images for: A noncoding RNA containing a SINE-B1 motif associates with meiotic metaphase chromatin and has an indispensable function during spermatogenesis
Source: PLoS One. 2017 Jun 28;12(6):e0179585. doi: 10.1371/journal.pone.0179585 (PMC5489172; doi:10.1371/journal.pone.0179585)

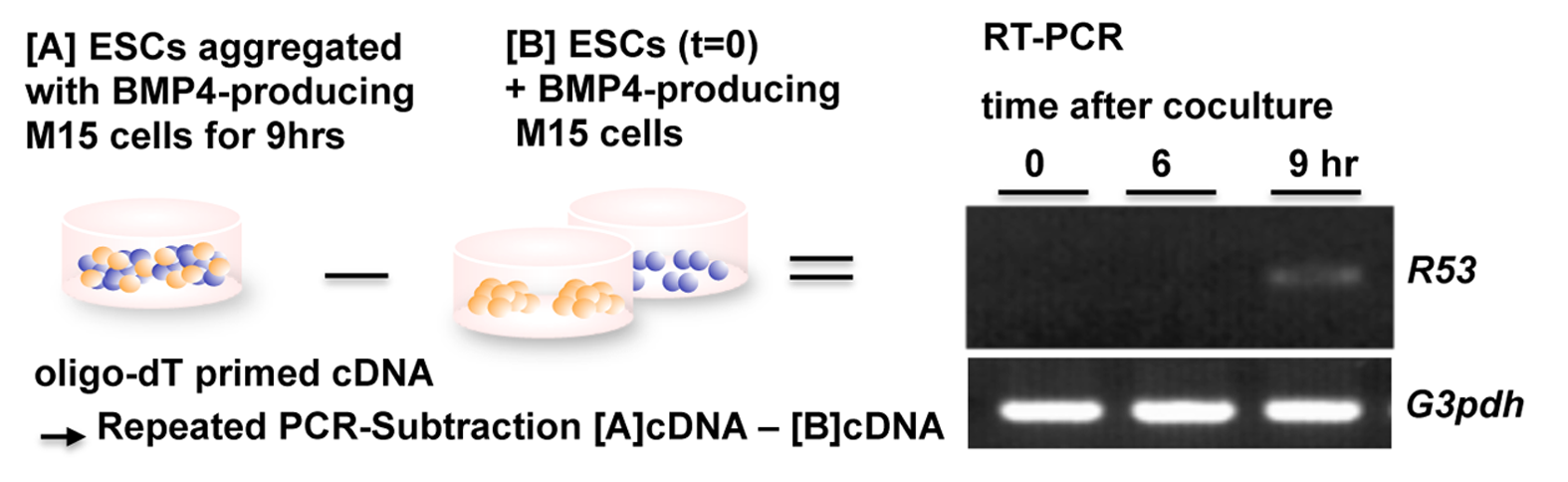

Supplement: S1 Fig — To isolate early response genes that were activated following germ cell induction from ESCs, repeated PCR subtraction was performed using cDNA prepared from a mixture of ESCs and BMP4-producing M15 cells versus cDNA from a suspension culture of mixed cells for 9hrs. RT-PCR using R53A (R53F and R53R) as primers to produce a R53 cDNA together with G3pdh products, which were used as a standard, revealed that R53 RNA expression became detectable 9 hr after the initiation of co-culture. (TIF) [file pone.0179585.s001.tif]

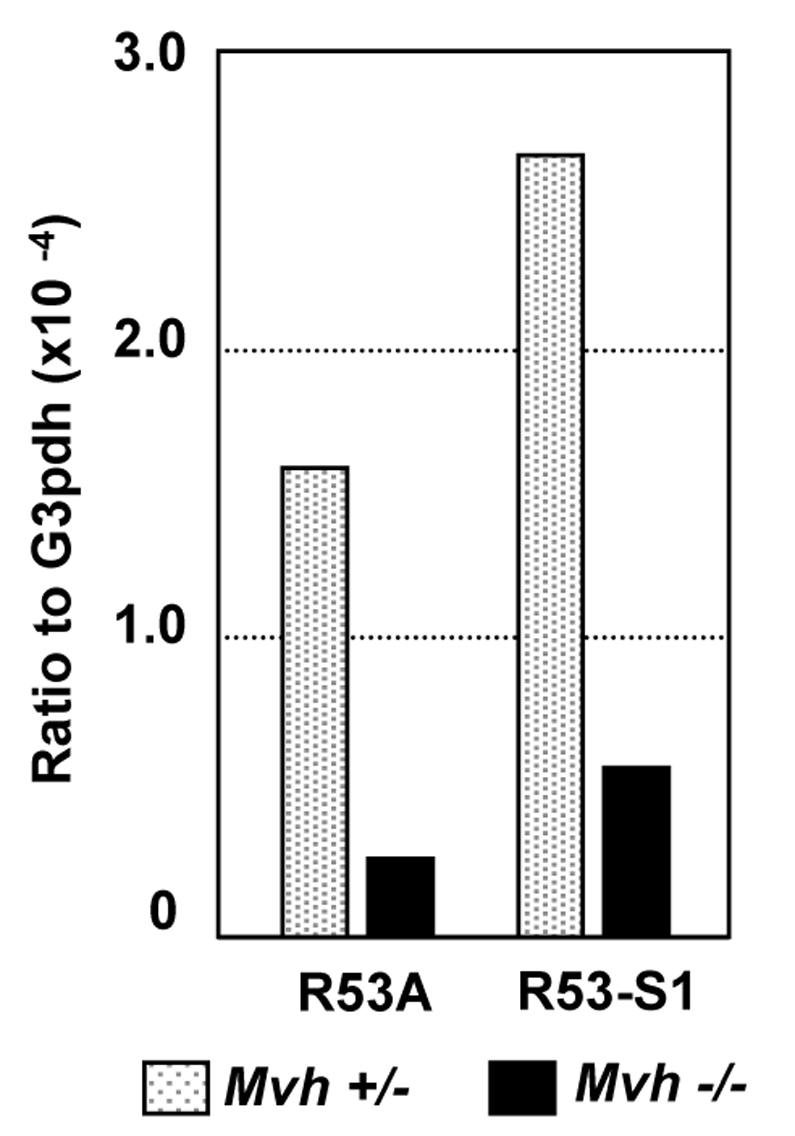

Supplement: S2 Fig — Single-stranded cDNA was prepared from adult testes (4-month-old) of Mvh heterozygote (+/-) and homozygote (-/-) mice. R53 expression was quantitatively analyzed with 2 pairs of primers, i.e., R53A (which detected the R53 cDNA, 587bp) and R53-S1 (which detected a R53-B1F element). The values are given as the relative ratios against the G3pdh gene expression (x10-4) and are average of three independent experiments. (TIF) [file pone.0179585.s002.tif]

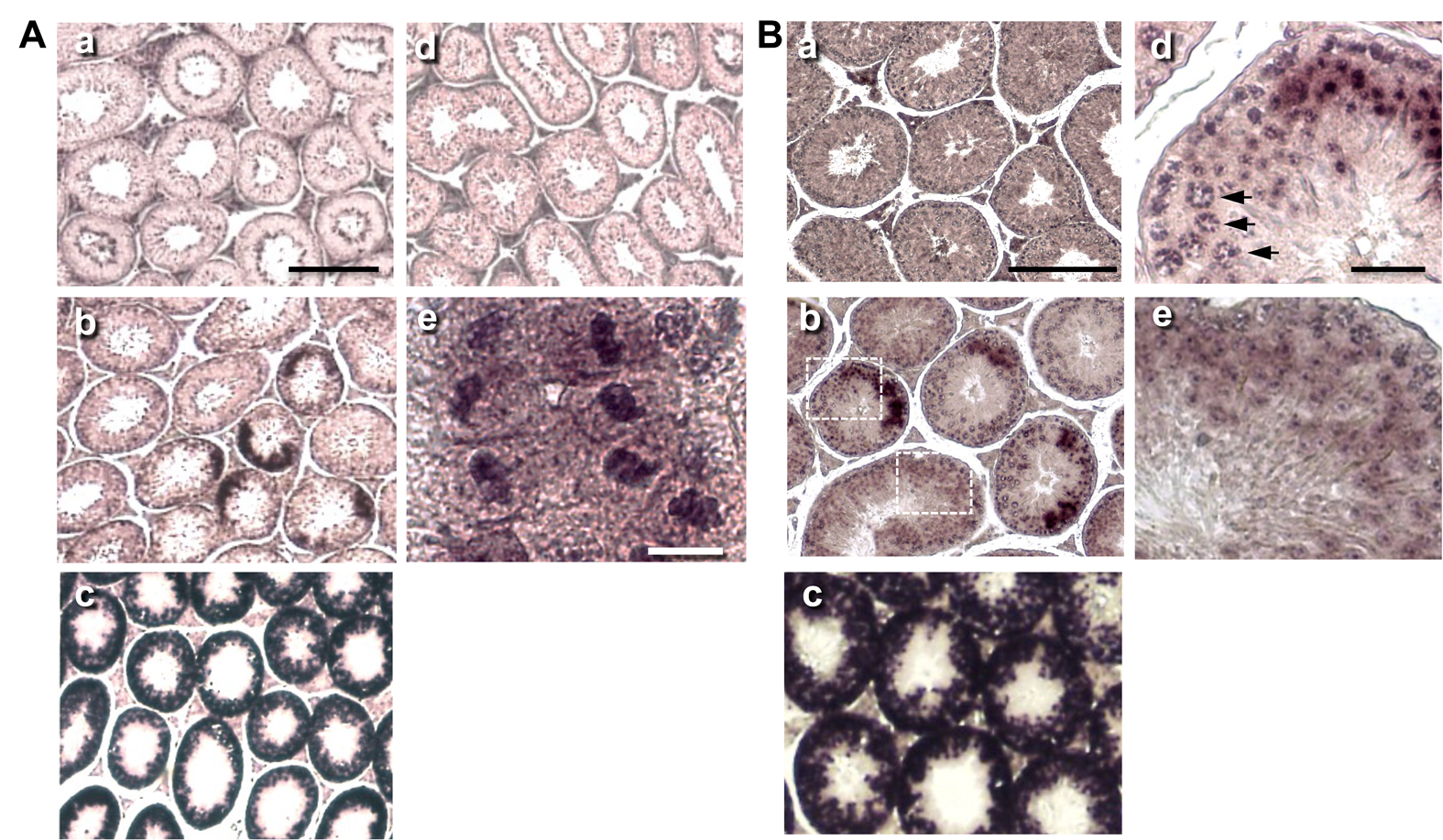

Supplement: S3 Fig — ISH against adult testis sections was performed using Dig-labeled 70-mer oligo DNAs, AS2 (antisense), S2 (sense) and ASpB1D (antisense) as probes (the positions are represented in Fig 2 and the sequences are listed in S1 Table and Fig 1). Hybridization and washing were performed in the same stringent condition and the colorization with an alkaline phosphatase reaction was performed at the same time for all the probes. A) ISH images of colorization for 4 hrs. (a) S2, (b) AS2 and (c) pB1D were used for probes, respectively. (d) Hybridization without probe that shows a background staining by AP- conjugated anti-Dig antibody. (e) High-magnification view of a portion of (b) showing the positive signals localized on the metaphase chromatin. The scale bar in (a) for (a—d) is 200 μm, and the bar in (e) is 20 μm. B) ISH images of clorization for approximately 16 hrs to strengthen the signals. (a) S2, (b) AS2 and (c) pB1D were used as probes. (d) and (e) High-magnification views of two outlined frames in (b). (d) and (e) are tubules at stage XII and stage V, respectively. Arrows in (c) indicate late pachytene spermatocytes. Compared with a staining image using sense probe in (a), the positive signals of R53 RNA become to be detectable on the pairing chromosomes of prophase spermatocytes and in the cytosol/nuclei of round spermatids. The scale bar in (a) for (a—c) and in (d) for (d, e) is 200 μm and 50 μm, respectively. (TIF) [file pone.0179585.s003.tif]

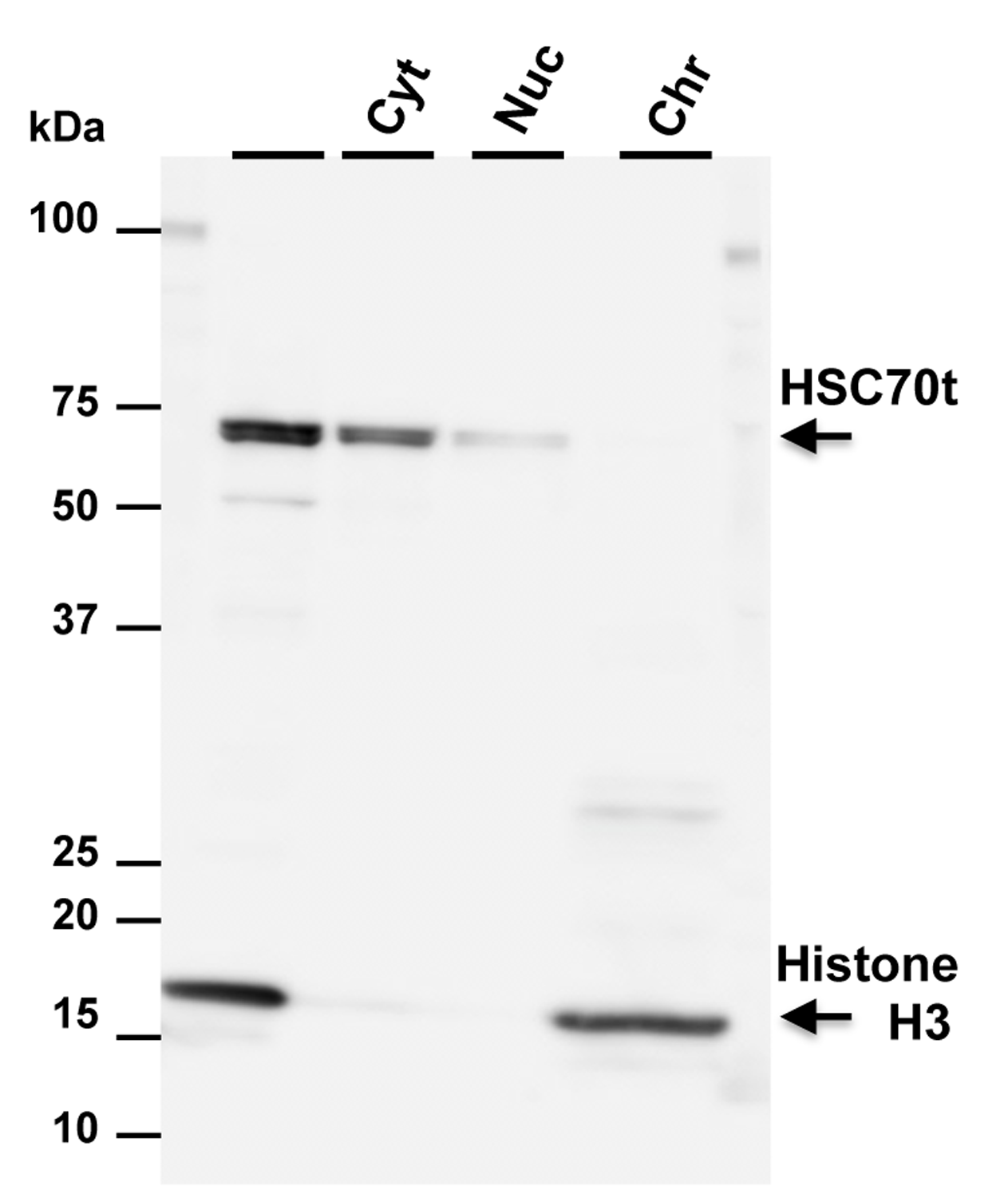

Supplement: S4 Fig — SDS-sample solutions were prepared from whole-cell extract and subcellular fractions (Cyt, Nuc and Chr) and a half volume of each solution was subjected to SDS-PAGE. The blotted membrane was reacted with a mixture of anti-HSC70t and anti-Histone H3 and visualized via a HRP reaction using anti-rabbit IgG secondary antibody. The antibodies used in this analysis are listed in S2 Table. (TIF) [file pone.0179585.s004.tif]

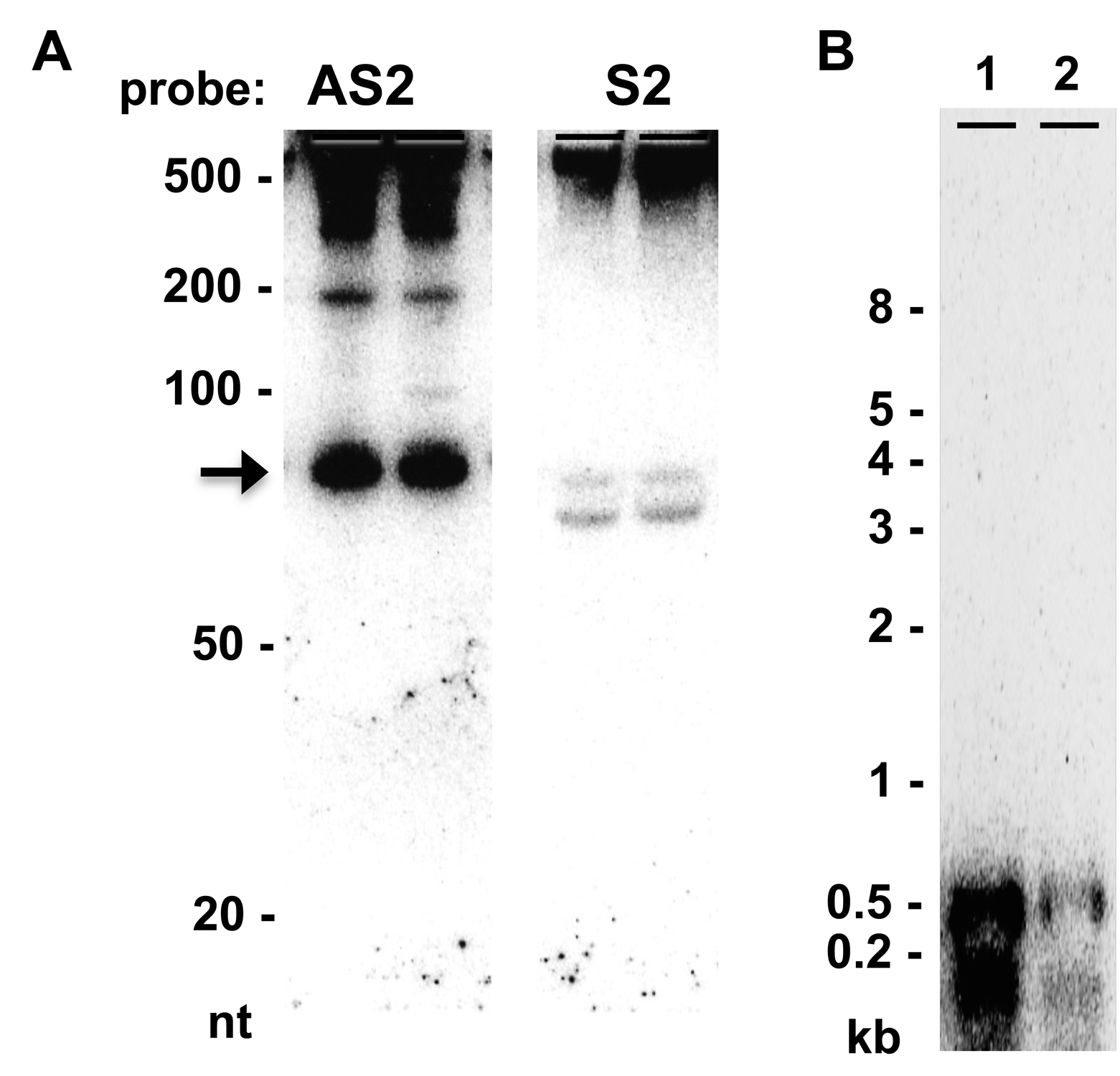

Supplement: S5 Fig — (A) A total of 10 μg of total RNA extracted from adult (4-month-old) testes was used for PAGE (duplicated in two lanes), and then two sets of electro-blotted membranes were hybridized with AS2 antisense or S2 sense probes. The arrow indicates the band of approximately 80 nt specifically hybridized with AS2 probe. (B) A total of 40 μg (lane 1) and 20 μg (lane 2) of total RNA prepared from adult testis (3-month-old) were electrophoresed using a 1.1% formalin-agarose gel. The blotted membrane was hybridized with AS2 antisense probe and then the hybridizing signals were detected as described in materials and methods. (TIF) [file pone.0179585.s005.tif]

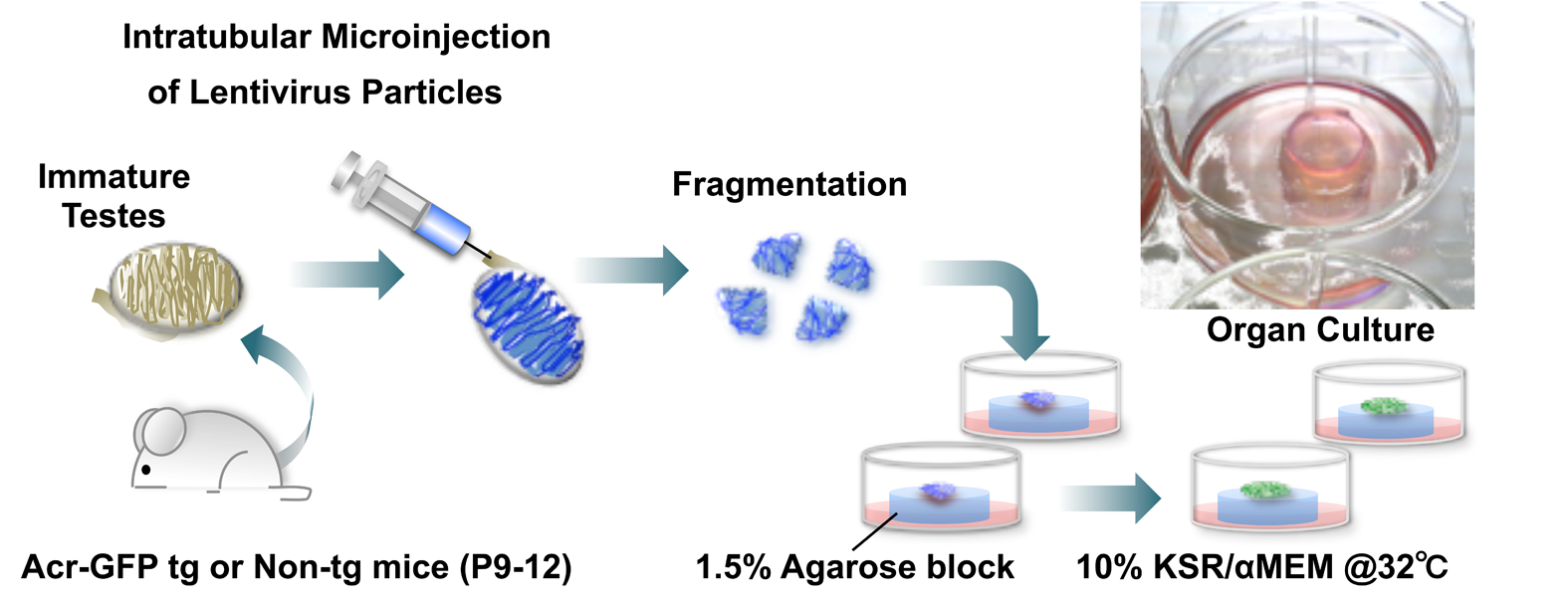

Supplement: S6 Fig — After intratubular injection of the lentivirus particles, the testes were fragmented and then positioned on the agarose blocks soaked in organ culture medium. (TIF) [file pone.0179585.s006.tif]

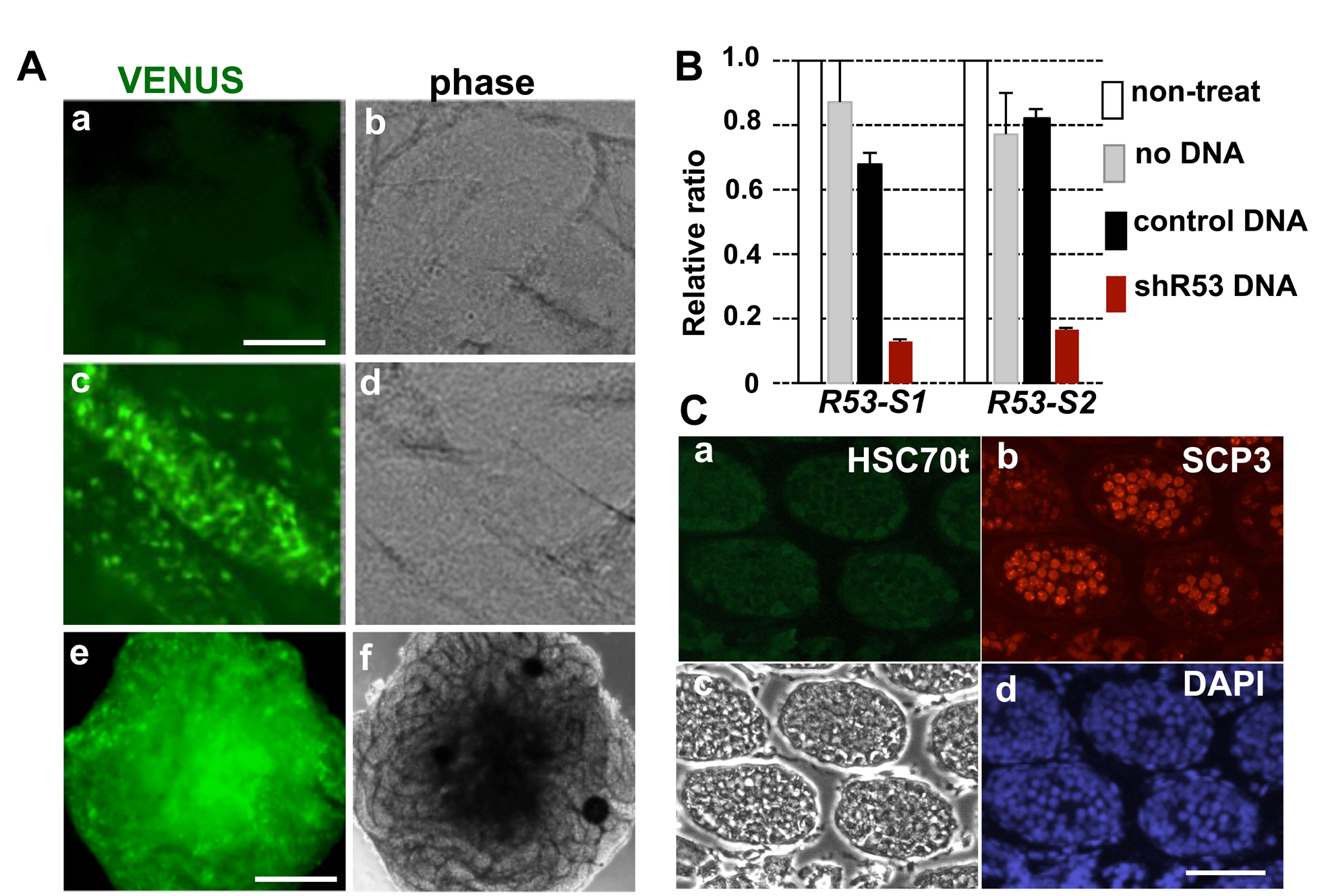

Supplement: S7 Fig — (A) The efficiency of lentivirus-mediated gene transduction into testicular tubules was examined with a lentivirus vector carrying a CAG-Venus gene. Non-treated and lentivirus-injected testes (from wild-type littermates at P11) were fragmented into 4 pieces and cultivated on agarose blocks. Four days later, non-treated (a, b) and lentivirus-injected (c, d) fragments were examined their Venus expression as shown in each phase contrast image. (e, f) Pictures of a 21 day culture of a lentivirus-injected fragment. The scale bar in (a) is for (a-d), and the scale bar in (e) is for (e, f), the bars are100 μm and 1mm, respectively. (B) The knockdown efficiency of shR53 RNA was first examined using a HEK293T cell line carrying a R53 forced-expression vector. Three days after transfection with the control lentivirus or the shR53 lentivirus vector DNAs, the single-stranded cDNAs were prepared from these transfected cells and non-transfected cells (without DNA) were used as the control. The R53 expression was quantitatively analyzed with two different primer pairs, i.e., for R53S1and R53S2, to detect the transcripts that contained an R53-B1F element. The values indicated are the relative values of the R53 expression level of the non-treated cells (set as 1.0) after standardization to the β-actin expression level. The error bars indicate the SEM (n = 6). (C) Sections of a P12 testis were double-stained with anti-HSC70t (a) and anti-SCP3 (b); Alexa Fluor-488 labeled anti-rabbit IgG and Alexa Fluor-568 labeled anti-mouse IgG were used as the secondary antibodies, respectively. The views of phase-contrast and nuclear staining with DAPI are shown in (c) and (d). The scale bar in (d) is 100 μm. (TIF) [file pone.0179585.s007.tif]

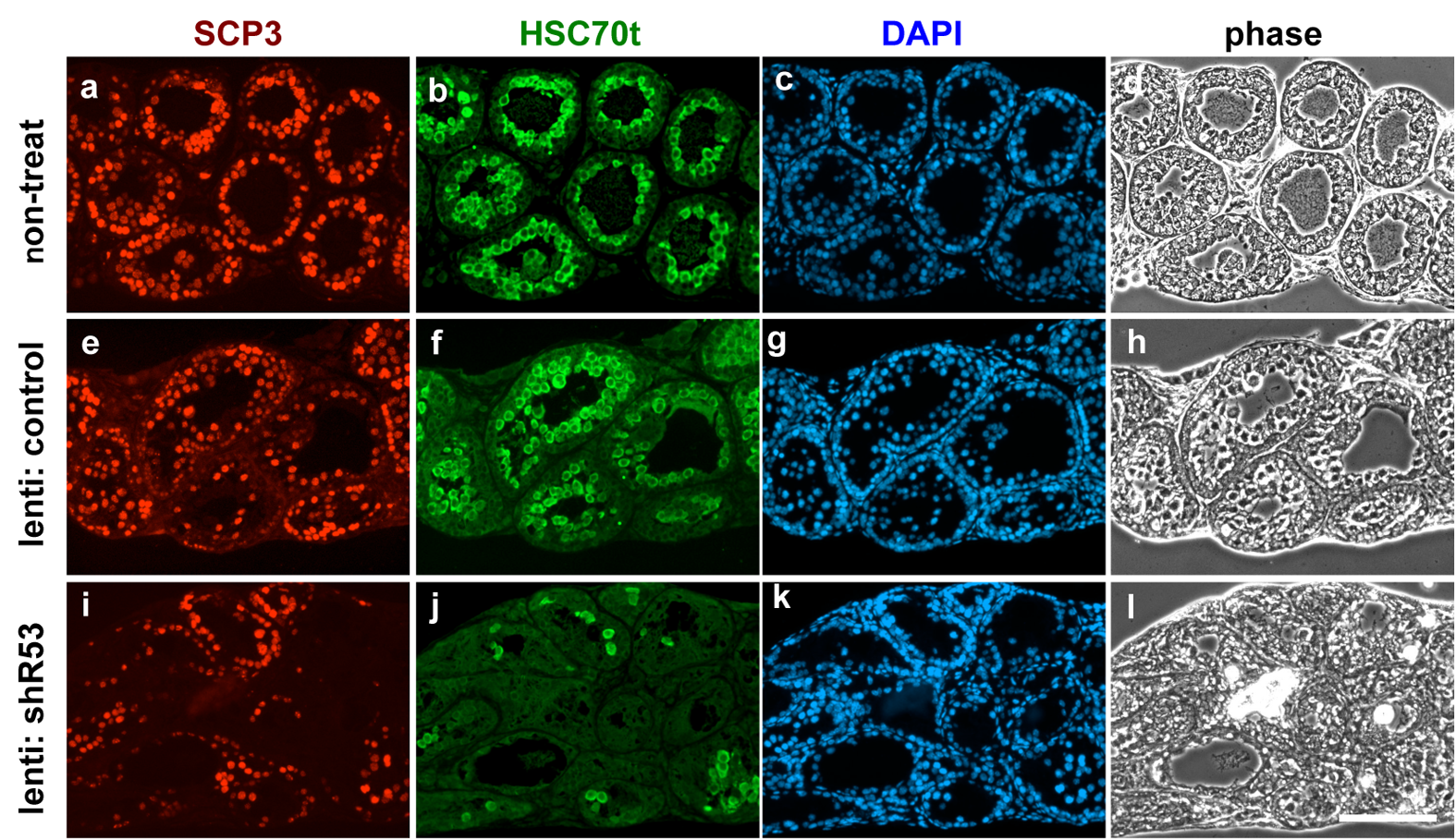

Supplement: S8 Fig — Fragments of immature testes (P9) from wild-type littermates were cultivated for 21 days. Histological sections from non-treated (a-d), control lentivirus-injected (e-h) and shR53 lentivirus-injected (i-l) fragments were stained with anti-SCP3 (a, e, i), anti-HSC70t (b, f, j) and DAPI (c, g, k), and the results are shown together with each phase contrast image (d, h, l). The scale bar in (l) for (a)-(l) is 100 μm. (TIF) [file pone.0179585.s008.tif]
