# Supplementary material for: A noncoding RNA containing a SINE-B1 motif associates with meiotic metaphase chromatin and has an indispensable function during spermatogenesis
Source: PLoS One. 2017 Jun 28;12(6):e0179585. doi: 10.1371/journal.pone.0179585 (PMC5489172; doi:10.1371/journal.pone.0179585)
Supplement: S1 Protocol — (DOCX) [file pone.0179585.s009.docx]

**SI Protocol**

**Repeated PCR subtraction**

The repeated PCR subtraction method was performed according to the method previously described by Mizuno *et al.* [46]. Briefly, total RNA was extracted from a mixture (1:1 ratio) of ESCs and BMP4-producing M15 cells and from cell aggregates of the mixture in a suspension cultured for 9 hr (10% FCS/ DMEM), as previously described [23]. Single-stranded cDNA was prepared from the total RNAs using an oligo-dT primer and a Superscript III First-Strand cDNA Synthesis Kit (Invitrogen) as per the manufacturer’s instructions. After subtracting 4 times, the resulting cDNA was subcloned into a pGEM-T Easy plasmid (Promega), and the sequence was determined. A pair of R53F and R53R PCR primers (S1 Table) was used to amplify the cDNA region of the *R53* transcript.

**Preliminary approval of the shR53 knockdown**

For preliminary experiments that were designed to confirm the knockdown efficiency of the shR53 vector, we performed the R53 knockdown using a HEK293T cell line carrying a *R53* forced-expression vector, pPyCAG-R53-IPacEGFPpA, which was made by replacement of the *R53* cDNA with the DsRed cassette of the original plasmid pPyCAG-DsRed-IPacEGFPpA [47]. The R53-HEK293T cells were transfected with either shR53 lentivirus or the control lentivirus vector DNA (2 μg per 2 ml culture) using Lipofectamine 2000 (Invitrogen). Three days later, each single-stranded cDNA was prepared from the total RNA, and *R53* expression was analyzed by qRT-PCR (S6 Fig).

**Western-blot analysis**

Protein samples prepared using a subcellular fractionation method were separated by SDS-PAGE (5-20% gradient gel). The electro-blotted membrane (Immobilon-XP) was immunoreacted with primary antibodies and visualized with the ECL Prime Western blotting Detection kit (GE Healthcare) containing secondary antibodies, Horseradish peroxidase (HRP)-labeled anti-rabbit IgG, and the Quant LAS4000-mini (GE Healthcare) in accordance with the manufacturer’s instruction. The antibodies used for immunoblotting detection are listed in S2 Table.

**Supplementary references**

1. Mizuno K, Tokumasu A, Nakamura A, Hayashi Y, Kojima Y, Kohri K, et al. Genes associated with the formation of germ cells from embryonic stem cells in cultures containing different glucose concentrations. Mol Reprod Dev 2006; 73: 437-445.
2. Nakatake Y, Fujii S, Masui S, Sugimoto T, Torikai-Nishikawa S, Adachi K, et al. Kinetics of drug selection systems in mouse embryonic stem cells. BMC Biotechnol. 2013; 13: 64-73.
