# Supplementary material for: A noncoding RNA containing a SINE-B1 motif associates with meiotic metaphase chromatin and has an indispensable function during spermatogenesis
Source: PLoS One. 2017 Jun 28;12(6):e0179585. doi: 10.1371/journal.pone.0179585 (PMC5489172; doi:10.1371/journal.pone.0179585)
Supplement: S1 Table — (DOCX) [file pone.0179585.s010.docx]

**S1 Table. Primers and probes used in this study**

| **Name** | **Primer Pair Sequence (5' to 3')** | **Use** |
| --- | --- | --- |
| R53A | R53F (forward): CTCAGGTACGTGTCTCCTTC　　　　 R53R (reverse): CGGCATCCTGCCATTAAGGG | RT-PCR |
| R53-S1 | forward: GGCTGAGGCAGGAGAATCAC　　　　　　　 reverse: AAGGGGTTGAGATCTGTAGG | qRT-PCR |
| R53-S2 | forward: CCGGAGGAACTGTATTAACG　　　　　　　 reverse: CGGCATCCTGCCATTAAGGGCTA | qRT-PCR |
| G3pdh | forward: GGTCATCATCTCCGCCCCTT　　　　　　　　 reverse: GCCAGTGAGCTTCCCGTTCAGC | RT-PCR |
| qG3pdh | forward: AAGGTCATCCCAGAGCTGAA　　　　　　　 reverse: ACAACCTGGTCCTCAGTGTAG | qRT-PCR |
| q*β-*Actin | forward: TATGGAATCCTGTGGCATCC　　　　　　　 reverse: ACATCTGCTGGAAGGTGGAC | qRT-PCR |
| qMvh | forward: GTATTCATGGTGATCGGGAGCAG　　　　 reverse: CAACAAGAACTGGGCACTTTCCA | qRT-PCR |
| qSycp3 | forward: ATGAATGTGTTGCAGCAGTG　　　　　　　 reverse: GCTCGTGTATCTGTTTGATTGC | qRT-PCR |
| qDmc1 | forward: TTTCAAGACATTGACCTGTTGC　　　　 　 reverse: TAAGCTTGTTGGCTGCCTCT | qRT-PCR |
| qTnp1 | forward: GAGAGGTGGAAGCAAGAGAAAA　　 　 reverse: CCCACTCTGATAGGATCTTTGG | qRT-PCR |
| qTnp2 | forward: GAAGGGAAAGTGAGCAAGAGAA　　　 reverse: GCATAGAAATTGCTGCAGTGAC | qRT-PCR |
| qPrm1 | forward: ACACAGGCGCTGCTTCGTAA　　　　　　 reverse: GTGATGGTGCCTCCACATTTCCT | qRT-PCR |
| qAcr | forward: TGTCCGTGGTTGCCAGGATAACA　　 reverse: AATCCGGGTACCTGTTGTGAGTT | qRT-PCR |
| qCasp9 | forward: TCCTGGTACATCGAGACCTTG　　　　 　 reverse: AAGTCCCTTTCGCAGAAACAG | qRT-PCR |
| qP53 | forward: CACGTACTCTCCTCCCCTCAAT　　　　 reverse: AACTGCACAGGGCACGTCTT | qRT-PCR |
| qB1D | forward: GCACGCCTGTAATCCCAGCTACT　　 　 reverse: AGAGACAGGGTCTCGCTATGTAG | qRT-PCR |
| qBC1 | forward: CGGTCCTCAGCTCTGGAAAA　 reverse: GGTTGTGTGTGCCAGTTACC | qRT-PCR |
| qXist | forward: AGATGGGGCATGAGGATCCT reverse: TCAGTGCCACTATTGCAGCA | qRT-PCR |
| qJ1C (genome) | forward: 5’- GCTGTTATAATACCCTGGTG reverse: 5’- AAGTCACTCAGGTACCGAAGG | qRT-PCR |
| **Name** | **Probe Sequence (5' to 3')** | **Use** |
| S2 | ACACATGGGAGGCTGAGGCAGGAGAATCACAAATTTGAGGCCAGCCTGGGTTACATAGTGAGTTTCAGAG (70 mer) | ISH, Northern |
| AS2 | CTCTGAAACTCACTATGTAACCCAGGCTGGCCTCAAATTTGTGATTCTCCTGCCTCAGCCTCCCATGTGT (70 mer) | ISH, Northern |
| ASpB1D | ACAGGGTCTCGCTATGTAGCCCAGGCTGGCCTCGAACTCAGCGATCCTCCTGCCTCAGCCTCCCGAGTGC (70 mer) | ISH, Northern |
