# Supplementary material for: A noncoding RNA containing a SINE-B1 motif associates with meiotic metaphase chromatin and has an indispensable function during spermatogenesis
Source: PLoS One. 2017 Jun 28;12(6):e0179585. doi: 10.1371/journal.pone.0179585 (PMC5489172; doi:10.1371/journal.pone.0179585)
Supplement: S2 Table — (DOCX) [file pone.0179585.s011.docx]

**S2 Table. Antibodies used in this study**

IHC: immunohistochemistry, WB: western-blot, Rb: rabbit, Ms):mouse, Gt: goat, Dn: donkey, Ch: chicken

| **Antibody (species)** | **Manufacturer (code or reference)** | **Use (dilution rate)** |
| --- | --- | --- |
| anti-HSC70t(Rb) | Self-made [44] | WB (1/4000), IHC (1/1000) |
| anti-MVH (Rb) | Self-made [45] | IHC (1/2000) |
| anti-SCP3 (Ms) | Abcam (ab97672) | IHC (1/1000) |
| anti-Histone H3 (Rb) | Abcam (ab1791) | WB (1/4000) |
| anti-GFP IgY(ch) | Aves LABs. Inc. GFP-1010 | IHC (1/1000) |
| [Alexa Fluor-488] anti-rabbit IgG (Gt) | Thermo Fisher Scientific (A-11034) | IHC (1/1000) |
| [Alexa Fluor-568] anti-mouse IgG (Gt) | Thermo Fisher Scientific (A-11031) | IHC (1/1000) |
| [Alexa Fluor-488] anti-chicken IgY (Gt) | Thermo Fisher Scientific (A-11039) | IHC (1/1000) |
| [HRP] anti-rabbit IgG (Dn) | GE-HealthCare (NA934VS) | WB (1/2000) |
